# Exploring Protein Dynamics Space: The Dynasome as the Missing Link between Protein Structure and Function

**DOI:** 10.1371/journal.pone.0033931

**Published:** 2012-05-11

**Authors:** Ulf Hensen, Tim Meyer, Jürgen Haas, René Rex, Gert Vriend, Helmut Grubmüller

**Affiliations:** 1 Theoretische und computergestützte Biophysik, Max-Planck-Institut für biophysikalische Chemie, Göttingen, Germany; 2 CMBI, Radboud University Nijmegen Medical Centre, Nijmegen, The Netherlands; Aberystwyth University, United Kingdom

## Abstract

Proteins are usually described and classified according to amino acid sequence, structure or function. Here, we develop a minimally biased scheme to compare and classify proteins according to their internal mobility patterns. This approach is based on the notion that proteins not only fold into recurring structural motifs but might also be carrying out only a limited set of recurring mobility motifs. The complete set of these patterns, which we tentatively call the dynasome, spans a multi-dimensional space with axes, the dynasome descriptors, characterizing different aspects of protein dynamics. The unique dynamic fingerprint of each protein is represented as a vector in the dynasome space. The difference between any two vectors, consequently, gives a reliable measure of the difference between the corresponding protein dynamics. We characterize the properties of the dynasome by comparing the dynamics fingerprints obtained from molecular dynamics simulations of 112 proteins but our approach is, in principle, not restricted to any specific source of data of protein dynamics. We conclude that: 1. the dynasome consists of a continuum of proteins, rather than well separated classes. 2. For the majority of proteins we observe strong correlations between structure and dynamics. 3. Proteins with similar function carry out similar dynamics, which suggests a new method to improve protein function annotation based on protein dynamics.

## Introduction

The Anfinsen experiment [1] showed that protein structure, in principle, is determined by its sequence. Later this conclusion was nuanced when chaperones, the amyloidal state, natively unfolded proteins, etc., were discovered, but the concept that sequence determines structure – and ultimately function –, is still generally valid. Indeed, sequence alignments have revolutionized taxonomy and have become invaluable tools to derive phylogenetic trees, to predict domains in proteins for which no structural information is available, and to identify functionally important residues.

New sequencing methods discover vast amounts of so far uncharacterized proteins and much effort is spent in the field of bioinformatics to improve existing and develop novel methods for sequence based function annotation [2]. Most of these methods are based on homology concepts. Simply speaking, if two proteins are homologs, they are likely to have highly similar structures and the same or similar functions. Unfortunately, sequences of homologous proteins with similar structure and function can diverge so far that their homology cannot be detected from their sequences alone. Chothia and Lesk showed in 1986 that the structure of a protein remains more conserved during evolution than its sequence [3], and subsequently Sander and Schneider quantified this relation [4].

Accordingly, prediction of protein function from sequence data alone is limited by this rather indirect and complex relationship between sequence and function, and reliable annotations require close homologues with over 40% sequence identity over large enough portions of the sequence [5]–[7], thus posing a fundamental limit to function prediction from sequence alone. BLAST [8] is by far the most widely used software for sequence similarity detection, and when BLAST fails fails to detect homology scientists tend to resort to PSI-BLAST [9], threading techniques [10], [11], hidden Markov models [12], or laboratory experiments.

**Figure 1:**
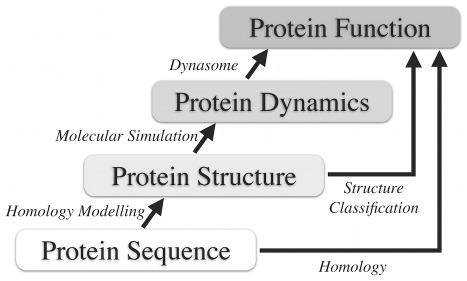
Schematic hierarchy of protein sequence, structure, dynamics, and function. Correlations between the various levels enable predictions. Here we explore the level of protein dynamics, and how it relates to structure and, respectively, protein function.

Structure can be seen as an intermediary between sequence and function, as exemplified in Fig. 1. Accordingly, in the absence of detectable sequence similarity, attempts have been made to infer function from structure similarities [13], and thus the classification of structures has become similarly important as sequence analyses for our understanding of protein function. Systems like CATH [14], SCOP [15], and DALI [16] provide a good overview of the protein structure universe. Indeed, the move from the sequence level to the structure level revealed more direct relations to protein function, and structure-based protein function predictions have proven more reliable [17].

These studies have, however, also shown that the relation between structure and function does, in the absence of sequence similarity, not permit reliable function predictions. Very different structures can have the same function (proteases, for example, occur in many branches of the classification trees of CATH, SCOP, and DALI) and very similar structures can have very different functions; the TIM-barrel fold, for example, has been observed with nearly all enzymatic functions known. As a result, purely structure-based protein function predictions have so far not been able to predict protein function beyond 30% reliability.

Most often, it is protein motion that is required for protein function (Fig. 1). If nothing can move, nothing can function. Perutz described the movements hemoglobin must undergo to fulfill its function shortly after the structure was solved [18], and Frauenfelder pioneered the field by flash photolysis experiments which revealed a hierarchical organization of protein motions - from thermal vibration to functional and collective conformational transitions [19] over 25 years ago. Detailed understanding on how dynamics leads to function is nevertheless still limited to few well-studied cases such as hemo/myoglobin or aquaporin, which selectively controls diffusion of water and small molecules through membranes [20]. Assuming that protein function is determined by protein motions more directly than by protein structure, we here decided to carry the move from sequence space to structure space one step further.

Such a general classification scheme for protein dynamics, similar to existing structure classifications, which captures the dynamics-function relationship, should also allow improved function prediction. Dynamics-based protein classification requires i) access to dynamics data of a representative set of proteins and ii) a similarity metric for dynamics of even structurally quite different proteins. Recent studies have, e.g., compared a particular protein in different environments [21]–[23], or similar proteins in a particular environment [21], [24], or the unfolding of a number of different proteins [25]–[30].

In this study, we carried out molecular dynamics simulations on a set of 112 proteins that represent a sufficiently large fraction of the ‘universe’ of known structures, and developed a systematic and unbiased methodology to quantitatively compare molecular dynamics simulations for very different proteins. To this aim, for each of the 112 trajectories, 34 dynamics observables were calculated, e.g. fluctuation amplitudes and frequencies, the eigenvalue spectrum of principal components etc. These have been chosen such as to characterize the many different aspects of protein dynamics to sufficient extent as to allow characterization of the dynasome and, taken together, yield a 34-dimensional vector for each protein. Each of these 112 vectors served to characterizes the dynamics of the respective protein, which is thereby represented as a point in the 34-dimensional ‘dynamics space’.

Subsequent analysis of the distribution and mutual distances of these 112 vectors revealed that 1) the universe of protein dynamics is covered by our subset of 112 proteins rather homogeneously, and in particular does not show pronounced clusters of proteins with highly similar dynamic behaviors, 2) that the two main characteristics that best describe the differences between the molecular dynamics simulations relate to protein thermodynamics and protein kinetics, respectively, and 3) that protein dynamics correlates remarkably well with protein function, allowing straightforward function prediction.

## Methods

### Approach and Concepts

The core problem of any classification approach is the choice of a proper metric, which discerns similar from different samples. Here, the main question was how to assess whether the dynamics of two different proteins are similar or not. For proteins with similar structure one might use amplitudes, relaxation times etc. of the motion of corresponding structural elements such as helices or loops. For proteins of similar size, principal component analyses of the motions of the backbone may provide quantitative information [31]. For any given pair of possibly quite unrelated proteins, however, there is not even much heuristics available which would allow to quantify the similarity of their dynamics, with the notable exception of two recently proposed methods from Micheletti [32] and Biggin labs [33]. Similarly, it is unclear how putative correlations to protein function can be detected and quantified.

In this study, we used 34 dynamics observables that have been selected such as to characterize the many different aspects of protein dynamics in a minimally biased fashion. Some of these quantities, such as fluctuation amplitude and frequencies, the eigenvalue spectrum of principal components, or the fluctuation of the radii of gyration, are widely known and routinely used. Others, such as the ‘entropy’ of the distribution of fluctuations within the protein or the roughness of the energy landscape that governs the principal modes, were developed here for the particular purpose of characterizing aspects of protein dynamics that we felt were not sufficiently covered by the established observables. Very much as for the study of sequence/structure relationships, the used structural observables (e.g., radius of gyration, helical content, packing etc) should not directly depend on the underlying sequence length, also all 34 dynamics observables were be normalized to avoid, as much as possible, any correlation to sequence or structural quantities.

### Protein Selection

Proteins were selected from the pdbfinder database [34] such as to cover a large fraction of known folds and structure classes (Table 1). We only considered wild-type proteins that were categorized monomeric by the protein quaternary structure file server (PQS) [35] and required a resolution better than 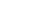 no ligands larger than 6 atoms, and no presence of metals other than Mg^2+^, Ca^2+^, K^+^, Na^+^, Zn^+^. Further requirements included a structure deposition date after 1987, an acceptable quality according to what check [36], absence of gaps larger than one amino acid, and structural stability during simulations. All structures passing these filters underwent visual inspection and were, if necessary, manually removed. Although, strictly speaking, 112 structures (Table S1) will not provide full coverage, we think that this number is large enough characterize the main features of the dynasome.

**Table 1:** Structure classes in the representative set of 112 proteins used in this study.

SCOP class	Number of proteins
*all*−*α*	12
*all*−*β*	33
*α*/*β*	27
*α*+*β*	30
*small*	10

### Simulation Setup and Ensemble Production

Protein structures were examined and corrected by what if [37], where the Whag [38] protocol was applied to correct for geometric errors in the backbone and side chains. Symmetry relaxed crystal waters that contact the monomer in the asymmetric unit cell were added. In case alternate atoms were present, the most abundant one was selected, and in case of equal occupancies the one with the alternate atom labeled A was used. Gaps in the structure of length one were filled as well as missing side chain atoms, which were placed by the rotamer library within what if [37]. The hydrogen bonding network was optimized as described in [39] and used to determine optimal rotamer angles and protonation states for Asn, His, and Gln residues. Aromatic groups with unphysical deviation from planarity were changed into planar conformation.

All simulations were carried out with the gromacs simulation suite [40], using the OPLS all-atom force field [41] and periodic boundary conditions. Proteins were solvated with a solvent shell of 1.1 nm TIP4P water and sodium and chloride ions were added (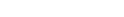). All systems were subsequently energy-minimized for 100 steps by steepest descent. The solvent was then equilibrated for 500 ps with positional restraints on the protein heavy atoms (force constant 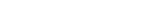). MD runs were carried out for at least 100 ns for each system generating an isothermic-isobaric (NPT) ensemble, with the protein and solvent coupled separately to a 300 K heat bath (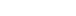) [42]. The systems were isotropically pressure-coupled at 1 bar (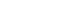) [42]. Application of the LINCS [43] and SETTLE [44] algorithms allowed for an integration time step of 2 fs. Short-range electrostatic and Lennard–Jones interactions were calculated within a cut-off of 1.0 nm, and the neighbor list was updated every 10 steps. The particle mesh Ewald (PME) method was used for the long-range electrostatic interactions [45], with a grid spacing of 0.12 nm. Coordinates were saved to trajectories every 200 fs.

### Trajectory Analysis and Dynamic Observables

All proteins were simulated for at least 100 ns, the first 20 ns were discarded as equilibration period, and the remaining 80 ns were analyzed. The 34 dynamics observables that were calculated from each of these trajectories, summarized in Table 2, fall into four groups i-iv:

**Table 2:** These 34 dynamics observables 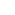 to 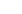 have been used to characterize the dynasome.

Index	Symbol	Description
1, …, 5	*λ*_1_, …, *λ*_5_	Eigenvalues 1, … 5
6	*m^λ^*	Slope of the middle third of the eigenvalue spectrum
7	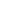	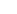 value of the fit to the eigenvalue spectrum
8,…,12	cos_1_,…,cos_5_	Cosine content of the principal components 1–5
13, 14, 15	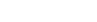	Goodness of fit of a Gaussian fit to principal components 1–3
16, …, 20	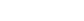	Friction constant derived from a fit to the autocorrelation function of principal components 1–5
21	*μ^γ^*	Measure of the average ruggedness of the energy landscape: Average slope of a linear fit to the time dependent eigenvalue spectrum *γ*.
22	skew*^γ^*	Skewness of the distribution of these ruggedness values (6) of each collective degree of freedom.
23	kurt*^γ^*	Kurtosis of the distribution of these ruggedness values.
24	*μ* ^RMSD^	Average root-mean square deviation from the X-ray structure
25	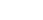	Standard deviation (% of mean) of the root-mean square deviation from the X-ray structure
26	*μ* ^RMSF^	Average residual fluctuations with respect to the average ensemble structure
27	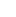	Standard deviation (% of mean) of the radius of gyration
28, …, 31	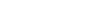	Standard deviation (% of mean) of secondary structure content: total, 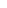 -helix, 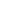 -sheet, turn
32	*μ* ^SAS^	Average solvent accessible surface
33	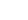	Standard deviation (% of mean) of the solvent accessible surface
34	*S_RMSF_*	RMSF entropy

#### i) Characterization of the eigenvalue spectrum of the proteins

Eigenvalues 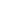 and eigenvectors 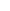 were obtained from diagonalization of the covariance matrix of C^α^ fluctuations, following the principal component analysis (PCA) protocol of Amadei et al. [31]. Eigenvalues were normalized to unit sum and the five largest eigenvalues were recorded as the first five (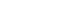) of the 34 dynamics observables listed in Table 2. Prompted by the observation that the central part of the eigenvalue spectrum resembles a power law [46], the region between 33% and 66% of the eigenvalue indices *i* was fitted to the function 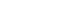 The fit parameter *b*, and the quality of the fit, quantified by the coefficient of determination (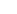), were used as observables 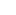 and 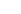


#### ii) Analysis of the Principal Components of the trajectory

Each of the *T* = 80′000 frames recorded in the 20 to 100 ns window were projected onto the first five eigenvectors 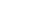 to obtain the essential coordinates 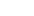 From these, as a measure for the extent of sampling, the cosine contents [46] of the first five principal modes were calculated as
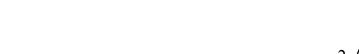
and recorded in observables 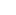 to 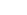 The distribution functions (PDF) of the first three essential coordinates were obtained by binning. From fits of Gaussian functions 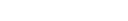 to these PDFs, 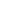 values were determined and recorded as 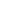 to 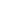


To gain insight into the time dependence of the dynamics of the 112 proteins, the fluctuations of the essential coordinates 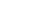 were described by a Ornstein Uhlenbeck process [47], i.e. by diffusion within a harmonic potential well. Accordingly, the autocorrelation function of this process, 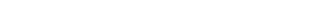 where 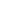 denotes the time interval between two frames, was fitted to the one obtained from the essential coordinates, 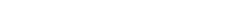 The fit parameters 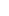 and 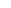 relate to friction and force constants of a harmonic oscillator. They were strongly correlated and we considered only the friction constants 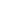 of the first five principal components 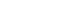 and used them as observables 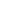 to 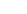


#### iii) Ruggedness of the free energy landscape

**Figure 2:**
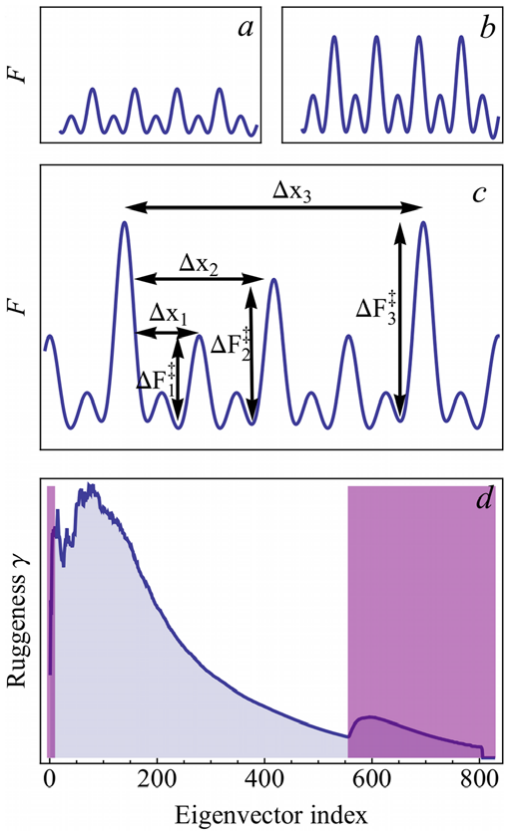
Illustration of the ruggedness parameter 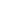 used as a descriptor in this study. **a)** – **c)**: Schematic rugged energy landscapes. The ruggedness of **a)** and **b)** is identical; although absolute barrier heights differ, the factor by which the barrier heights increase with their distance along the conformational coordinate is the same. In contrast, the energy landscape shown in **c)** is characterized by a larger ruggedness. **d)** **A typical ruggedness profile** of a protein is characterized by a steep increase at small eigenvector indices and a subsequent smooth descent to a characteristic minimum. The ruggedness of each eigenvector is described by 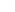 and the characteristics of the respective ruggedness profile (average ruggedness, skewness, and kurtosis) are used as descriptors 21, 22, and 23, respectively (Table 2). For the computation of these ruggedness descriptors, the first 10 eigenvectors and all eigenvectors beyond the characteristic minimum (purple shaded areas) were omitted.

As a further probe of protein dynamics we considered what we termed the (one-dimensional) ruggedness 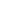 of the underlying free energy landscape. To that aim, we described the protein dynamics, projected onto the individual PCA eigenvectors in terms of diffusive motion within a potential that is formed by a hierarchy of energy barriers (Fig. 2 [48]). As sketched in the figure, this hierarchy is characterized at each tier by barrier heights 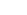 and an average distance 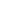 between the barriers of that height. As can also be seen, we assume the barrier heights to increase logarithmically with their mutual distances, i.e., 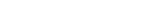 with a barrier height increment 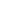 and a unit length *L*, below which we assume free diffusion with a diffusion coefficient 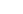 Hence, as indicated in Fig. 2a, b and c, the ruggedness as defined for the present purpose does not measure the barrier heights as such (a and b), but rather, how fast the barriers grow with increasing mutual separation (c).

As a result, the effective diffusion constant 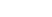 for protein motion within such hierarchical landscape decreases with the time scale *T* at which diffusion is monitored, and is governed by the rate limiting – i.e., largest – barrier 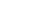 that is overcome by the system at this time scale, 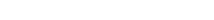 where 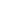 is the diffusion constant in the absence of barriers.

Vice versa, observation of the mean square distance 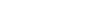 travelled by the protein as a function of trajectory length *T*, hence, provides information on the ruggedness of the underlying hierarchical energy landscape. Combining the above equations, and assuming 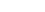 yields the power law
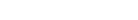
i.e. the eigenvalues 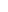 obtained from diagonalizing the covariance matrix of a trajectory of length *T* increase with trajectory length according to the power law 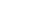. Hence, 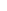 is obtained from the respective exponent 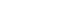. As should be expected, for free diffusion, this exponent is one, whereas for increasing ruggedness, diffusion ceases, and the exponent tends towards zero.

Using the above power law, the respective ruggedness of each of the 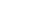 eigen-modes of each protein was determined from the (average) slope 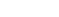 of the mean squared distance 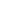 (obtained from the respective eigenvalue of a PCA) as a function of used trajectory length *T*, both in logarithmic representation. Accordingly, covariance matrices and their eigenvalues 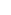 were calculated for 20 logarithmically spaced time windows ranging from 1 to 10 ns. For each window size, eigenvalues were averaged over 20 uniformly distributed trajectory parts to reduce statistical fluctuations. Fig. 1d shows as a typical ruggedness profile the values obtained for all 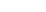 eigen-modes. Three observables were defined to characterize the overall shape of these ruggedness profiles, namely its average value 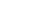 as well as the skewness (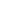) and kurtosis (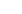). Because the dynamics of the largest eigen-modes is characterized already explicitly by other descriptors such as autocorrelation functions, the respective first 10 ruggedness values were excluded (left purple rectangle). Similarly, the fastest ca. 30% of the eigen-modes were also excluded (in the figure separated by the gap at eigenvalue 555, right purple rectangle), as these arise from essentially harmonic bond vibrations which are very similar for all proteins considered and, therefore, are not expected to provide additional information on their dynamics.

#### iv) Atomic fluctuations

The time-averaged root mean square deviation (RMSD) from the crystal structure 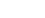 (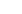) and its standard deviation relative to the mean 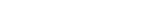 (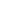) were calculated to quantify the average deviation from starting conditions. The overall flexibility of the protein was described by the RMS fluctuation with respect to the average structure (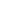), and *breathing* motions were quantified via the standard deviation of the radius of gyration 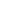 from its mean value (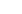). Secondary structure contents were determined over time using the Kabsch and Sanders algorithm [49] implemented in the ptraj program [50]. Relative fluctuations about the mean content were calculated for the total secondary structure 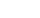 (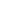) and the secondary structure elements a-helix 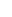 (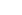), b-sheet 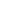 (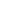) and turn 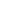 (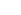). Solvent accessible surface area was calculated along the trajectory using naccess [51] with 1.4 Å probe radius, the mean polar solvent accessible surface (SAS) 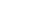 and 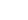 were used as observables 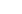 and 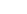


To describe the degree of localization of flexible regions in the protein we calculated the root mean square fluctuations (RMSF) 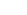 for each residue *i* of the protein using the ptraj program [50]. The resulting flexibility profile was characterized by its average 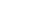 value and the entropy 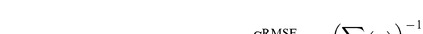 of the distribution, values were recorded in 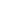 and 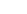


### Structure Analysis and Structure Observables

Similar to the 34 dynamics observables we defined a set of 24 non-redundant observables, listed in Table 2, that characterize protein structure. Structures of the 112 proteins were retrieved from the protein data bank (PDB) and missing atoms were added from the amber residue libraries using the program tleap [50]. Structures were then energy minimized in 100 steepest-descent steps with 25 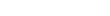 restraints on heavy atoms using the sander program from the amber10 package [50]. Observables were then calculated for each structure. Radii of gyration and moments of inertia along the three principal axis (g_gyrate [52]) 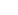 to 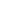 were calculated to characterize the overall shape of the protein. Further, the overall charge distribution was characterized by the proteins principal dipole moments, calculated using the g_dipoles program of gromacs and recorded in 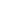 to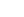 because imbalances in charge distribution are often associated to function [53]. Secondary structure content was determined as described above and the numbers of residues in helix, sheet, coil, and g-turn conformation, respectively, were recorded as structural observables 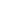 to 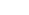


Intramolecular contacts were counted for non-neighboring (7 residues distance in sequence) residues where the 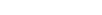 (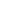); contacts were considered hydrophobic if both residues are of A, I, L, M, F, P, W, or V (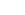). Hydrogen bonds were annotated using standard criteria 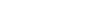 and 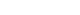 and counted (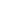). Total and hydrophobic solvent accessible surface areas were calculated using naccess [51] and recorded in 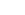 and 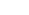


To describe protein topology, i.e. the non-local contacts, we generated for each protein its residue adjacency matrix with entries for all residues with at least two atoms closer than 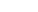 The matrix defines a network where residues are nodes and connections are drawn between adjacent residues [54], which we characterized by its average path length 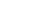 cluster coefficient 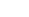 and network radius 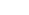 The number of hydrophobic, hydrophilic basic, acidic, proline, and lysine residues in the sequences were counted to characterize the chemical composition of the proteins, and were recorded as structure observables 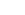 to 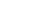


### De-correlation of Observables

Some of the dynamics (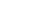) and structure (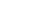) observables listed in Tables 1 and 2 were found to correlate markedly with sequence length. Because such correlation would, indirectly, introduce unwanted sequence information into the dynamics and structure observables, these were removed. To that aim, all observables for which correlations were detected were fitted to an appropriate model of the observed length dependence. After subtraction of the fit function, the now sequence-length de-correlated observables were normalized to zero mean and unity standard deviation. All fit functions are described in Table S2 and the pairwise correlations of the processed observables are displayed in Fig. S1.

### *k*-means Partitioning of the Dynasome

Clusters in the population of the 34-dimensional dynamics space were identified using a *k*-means algorithm, which iteratively minimizes the sum of distances of dynasome vectors to cluster centroids [55]. As initial guess, the location of the cluster centroids was chosen randomly. From 5000 runs with random initial conditions, the one with the smallest squared distance sum was used for subsequent analysis.

### De-correlation of Structure-dynamics Similarities

To assess to which extent structural similarity of proteins correlates with their dynamics similarity (Section ), the Euclidean distance 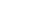 in structure space of all protein pairs 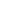 was plotted vs. their respective distance 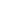 in dynamics space. The resulting plot was compared with a randomized reference data set, for which all correlations of 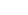 and 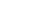 were eliminated by randomly permuting the 6216 pairs 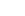 for 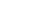 with respect to 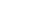 as sketched schematically in Fig. S2. As can be seen, this procedure eliminates all correlations between 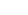 and 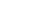 while preserving their individual distributions.

### Graphs of Mutual Adjacencies in Dynamics Space, Structure Space and Combined Space

The fine structure of the dynasome was represented as a graph of mutual adjacencies in dynamics space (see main text). First, adjacency matrices were obtained by identifying the *k* nearest neighbors of each protein in a *d* dimensional subspace of the 34-dimensional dynamics space, using the mathematica 7 *Nearest* function. For the visualization of the graph of the resulting adjacency matrix, we used the *GraphPlot* function with the spring electrical embedding option and the repulsive force power option set to −1.

Parameters *k* and *d*, required to calculate the adjacency matrix, were chosen as follows. For each pair 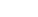 of 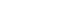 and 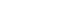, the adjacency matrix was computed as described above, the graph of this adjacency matrix was partitioned using the *CommunityStructureAssignment* module of mathematica, and the partitioning was quantified using the community modularity *C* (Fig. S3). For further analysis, the pair 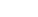 yielding the best partitioned graph was selected for further analysis (black points in Fig. S3).

### Assignment of Functional Classes

Proteins were assigned functional classes according to uniprot [56]. Table S1 lists the function class assigned to each protein. Poorly covered functional classes were collected as “Other” and “Other Enzymes” and not used for function prediction.

## Results and Discussion

### Generation of Dynasome Observables

We picked (cf. Methods) a set of 112 soluble, single-domain proteins from the protein database (PDB) [57], such that all structure classes were about equally represented. For each protein, explicit solvent all-atom molecular dynamics simulations of 100 ns length were carried out (cf. Methods) to sample the proteins native state dynamics at picosecond to 100 ns timescales. From each of the obtained trajectories, we calculated 34 observables, some of which specifically devised for this study (see Table 2). The combination of these provides a comprehensive characterization of the dynamics of each of these 112 proteins at time scales between picoseconds and 100 ns.

In this 34-dimensional “dynamics space”, spanned by the 34 observables, each protein is thus represented as a vector, and proteins of similar dynamics appear as nearby points in this space. We will refer to the whole “point cloud” of all proteins as the dynasome. Subsequently, we will investigate the properties and structure the dynasome.

### Few Collective Dynasome Descriptors Describe Most of the Dynasome

**Figure 3:**
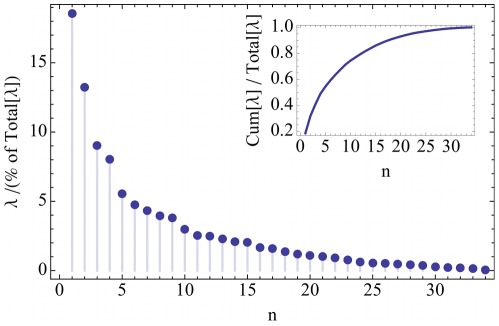
Eigenvalue spectrum of the collective dynamics descriptors Eigenvalues 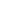 are given as fractions of the sum of all eigenvalues. The inset shows the cumulative distribution.

What are the most important dynamics features of that distinguish the 112 proteins from each other? To address this question, we carried out a principal component analysis (PCA) of the dynasome. Each of the resulting 34 eigenvectors constitutes a collective descriptor consisting of a linear combination of the 34 observables introduced above, very much as normal modes are linear combinations of individual atomic displacements [58], [59]. We refer to these linear combinations as dynasome descriptors. The eigenvalue profile (Fig. 3) shows that relatively few of these dynasome descriptors suffice to describe a large fraction of the dynamics seen in our protein set, e.g. the first 15 collective descriptors explain 80% (Fig. 3 inset) of the diversity of the dynamics of the examined proteins.

Notably, already the first two dynasome descriptors explain more than 30% of the dynamics variation seen in our protein set (Fig. 3). Table 4 (columns 1 and 2) lists those dynamics observables that contribute most to these two first descriptors. As can be seen, descriptor 1 contains the average deviations from the X-ray structure 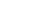 (entry 1) and from the ensemble average 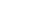 (entry 2), respectively, as (entry 3). All these observables characterize the magnitude of atomic fluctuations. The next two important observables in dynasome descriptor 1 are the average ruggedness 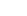 and skewness of the ruggedness spectrum (cf. Fig. 2). Their contribution (7%) to descriptor 1 is marked with (−) in Table 4, indicating anti-correlation of these two observables to the dynasome descriptor and reveal an interesting correlation: normally, fluctuations tend to be small for proteins for which the dynamics of the essential modes is governed by a rugged free energy landscapes (high skewness 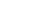 combined with high average ruggedness 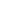). In contrast, large deviations from the X-ray structure are seen for relatively smooth energy landscapes (low 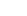) or if large-scale modes proceed along relatively smooth pathways compared to the small-scale high-frequency modes (low 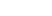). Strikingly, all the observables that dominate the most essential dynasome descriptor quantify ensemble properties.

The second dynasome descriptor is composed mainly of the friction coefficients of the diffusion along the first four (protein) eigenvectors 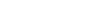 (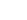 contributes 6% and is thus not listed in Table 4) and the Gaussianity of the proteins’ first principal component. In contrast to the first descriptor, these observables describe the time evolution of the global, collective motions of the systems, i.e. relate to kinetics. The correlation between friction coefficients at slow motions and deviations from Gaussianity reflects the frequent observation that slow degrees of freedom tend to be anharmonic.

It is a remarkable result that purely from an analysis of which observables contribute most to the dynamics diversity of the 112 selected proteins, and without any further input or bias, the above two dynasome descriptors were able to identify and distinguish ensemble properties (thermodynamics) from dynamics properties (kinetics).

**Figure 4:**
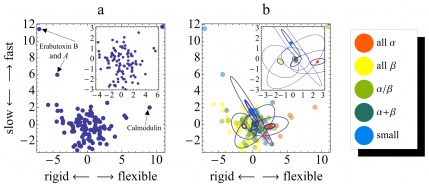
Projection of the dynasome onto descriptors 1 and 2. Each point represents one protein. **a)** Protein dynamics as described by dynasome descriptors 1 and 2. The axes labels indicate which dynamics properties are mainly described by the respective descriptor. The inset focuses on the lower left region. **b)** same projection as in **a)**, colored according to SCOP structure classes (see legend). Ellipses indicate the distributions of structure classes; Large thin ellipses denote standard deviations of the distributions, small thick ellipses the standard deviations of their mean.

**Figure 5:**
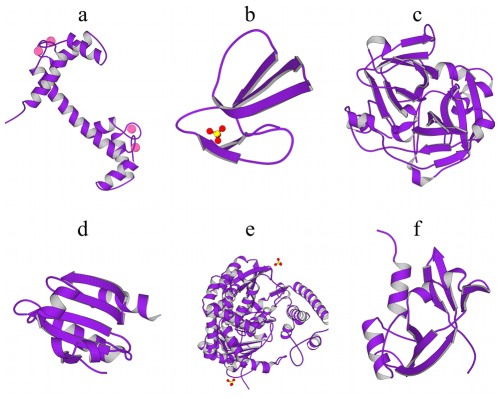
**Selection of six of the 112 proteins included in this study:** **a)** Calmodulin (PDB code 1OSA [71]), **b)** Erabutoxin B (PDB code 3EBX [72]), **c)** Achromobacter protease I (PDB code 1ARB [73]), **d)** Thioredoxin-2 (PDB code 1THX [74]), **e)** superantigen (PDB code 3SEB [75]), **f)** angiogenin (PDB code 1AGI [76]). Pictures were generated using MolScript [77].

A few typical examples shall illustrate how these two main collective dynamics descriptors serve to characterize the dynamics of particular proteins. Fig. 4a shows the distribution of the 112 proteins in the plane spanned by the dynamics descriptors 1 and 2. The axes labels indicate the type of dynamics, as summarized above, described by the respective descriptor. As a first example, calmodulin (Fig. 5a) is one of the most flexible structural proteins known to date and thus shows up as an outlier on the right of Fig. 4a. Calmodulin exhibits very large overall deviations from the crystal structure (reflecting its flexibility) and samples a very smooth energy landscape of extraordinarily low average ruggedness 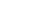 These two aspects are described by the dynasome descriptor 1 (*x* axis). The second dynasome descriptor (*y* axis) shows that the timescales on which Calmodulin dynamics take place, described by the friction constants 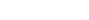 of diffusion along the first five eigen modes, are not unusual.

As a second example, the neurotoxins Erabutoxin A and Erabutoxin B (Fig. 5b) are both characterized by extremely flexible and fast moving loops held, tethered to a rigid core and stabilized by sulfide bridges. Both are outliers in the upper left part of Fig. 4a. The fact that fast and low amplitude motions dominate these proteins is revealed by very high average ruggedness 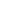 and large friction constants  as described by dynasome descriptor 2.

### Proteins Do not Fall into Well-separated Dynamics Classes

An interesting observation from Fig. 4a is that the projections on the first two dynasome descriptors show a rather continuous distribution without significant substructure. In light of the seemingly obvious clustering of protein structures that the reader may have in mind, this result is unexpected and will need careful discussion.

Before addressing this question in more detail, however, we investigated the extent of the structural classification reflected in the dynamics space. Fig. 4b shows the same projections as in a) with color codes indicating the SCOP structure class. Different structure classes tend to accumulate in different regions of dynamics space. 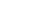 proteins are, for example, predominantly found on the right, whereas most 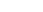 proteins are found to the left. 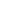-proteins overlap significantly with 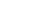 but are shifted slightly towards the bottom. *Small* proteins cover a large range from the upper left to the right. The standard deviation of the distributions of proteins of each SCOP class (large ellipses in Fig. 4b) show that the distributions overlap significantly. In contrast, the centroids of the different classes (centre of the ellipses) assume significantly different positions in dynamics space, as documented by the standard deviations of the mean (small circles).

We conclude that, on average, the dynamics of proteins described by the first two dynasome descriptors show a certain correlation to protein structure. The fact that the dynamics distributions of different structure classes overlap suggests, however, that there is no simple on-to-one mapping between protein structure and dynamics. Therefore, analysis of the dynasome should reveal additional information beyond that already contained in the protein structure.

Note that the above result of overlapping SCOP classes in dynamics space (Fig. 4) might also be a consequence of projecting 34-dimensional data onto two dimensions that account for slightly more than 30% but miss 70% of the overall dynamics features. If that was the case, then a non-hierarchical *k*-means clustering (methods) with a squared Euclidean metric in the full 34-dimensional space would reveal any internal structure – in particular, clusters – that might have been lost in the projection.

*k*-Means analyses with 1 to 10 cluster centers have been performed, but the analysis of the resulting clusters in terms of connectivity and variance [60] did not reveal any marked minimum (Fig. S4), which confirms that the absence of apparent clusters in Fig. 4 is not a projection artifact. Hence, also full space analysis did not reveal any natural partitioning, which agrees well with the visual inspection of Fig. 4.

Next, *k*-means clustering served to quantify possible correlations between SCOP and dynamics space. To that aim, we determined the overlap between the SCOP classes and the classification obtained by k-means clustering. First, to obtain better statistics, we considered only proteins belonging to the SCOP classes 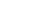 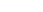 and 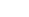 Figure 5a shows the distribution of these three SCOP classes into the three partitions of dynamics space identified by *k*-means clustering. As can be seen, more than 80% of the 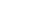 proteins are found in the first cluster, which contains less than 20% of 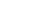 and less than 30% of the 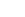 proteins, respectively. The second dynamics cluster, in contrast, contains almost 90% of all 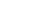 proteins and almost 70% of the 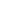 proteins, but less than 15% of the 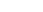 proteins. Obviously, α and 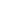 proteins are separated well in the full dynamics space, whereas 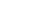 and 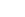 proteins overlap markedly, and to a similar extent as in the two-dimensional projection (ellipses in Fig. 4b).

**Figure 6:**
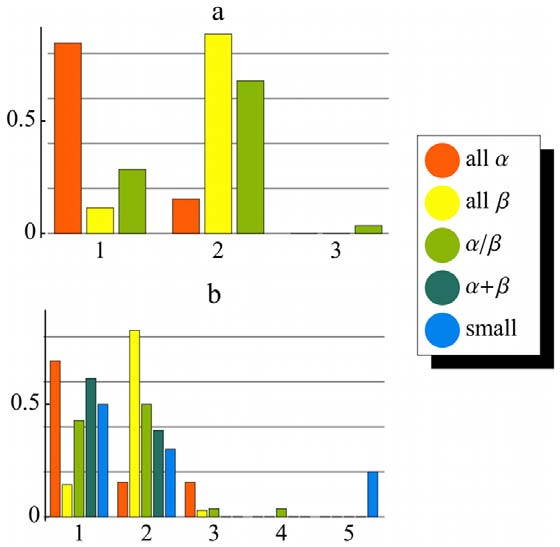
Recovery of structural classes from dynamics. Distribution of three **a)** and all five **b)** SCOP classes (colors) onto partitionings of the dynasome (1…5) obtained from k-means clustering. Bar heights denote the fraction of proteins of each structure class found in each partition.

Next, we considered all five SCOP structure classes and determined their distribution into a partitioning obtained from a k-means clustering for five classes. As can be seen from Fig. 6b, a similarly pronounced separation between 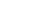 and 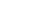/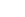 is obtained (Fig. 6a), whereas almost all a + b and *small* proteins can, purely on the basis of their dynamics fingerprint, not be well distinguished from 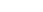 proteins.

Overall, our 112 sample proteins seem to populate dynamics space rather uniformly, without marked clusters or sub-families. Nevertheless, as was already visible in the two-dimensional projection (Fig. 4), the known structural classes tend to accumulate in different regions in dynamics space. This observation shows that structural classes, e.g., 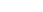 and 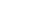 can be distinguished purely from their dynamics fingerprints. Also from this analysis, the remarkably large but not strict correlation between structure and dynamics points to additional information (or noise) that may be contained within protein dynamics but not within structure alone.

The finding that proteins are continuously distributed in dynamics space was actually quite unexpected. Several mechanisms might, alone or combined, explain our findings: First, the SCOP structure classes used here as a reference might suggest a much clearer partitioning of the structure space than would be obtained from an approach not based on discrete descriptors such as secondary structure class, which unavoidably implies a certain partitioning. A number of recent studies [61]–[63] indeed yield less pronounced partitioning suggesting that this effect might actually play a role. Our own structure-based analysis discussed further below provides further support for this possibility. Alternatively, the protein distribution in dynamics space might become ‘blurred’ with respect to that in structure space by the fact that already slight structural changes might imply quite different dynamics. We will quantify this possibility, referred to as ‘adjoint dynamics’ further below. Vice versa, similar dynamics patterns might arise from quite different structures (‘disjoint dynamics’). We intentionally refrained from the use of the more suggestive terms ‘convergent’ and ‘divergent’ to avoid any direct evolutionary interpretation, which would not be supported by our approach. As a third option, and despite our efforts to cover many different aspects of protein dynamics, we cannot completely rule out the possibility that the 34 dynamics observables we have chosen simply do not suffice to provide a sufficiently complete picture of the dynasome to be able to detect an existing partitioning. To test for this possibility, we repeated the above analysis using different subsets of these dynamics variables, without significant changes of the obtained partitioning.

### Protein Structure Classes Overlap Significantly

We thus asked which of these mechanisms is actually at the root of the observed continuous distribution in dynamics space. The first question we addressed was whether or not natural structure classes are evident if a similarly systematic approach as used above for protein dynamics is applied to protein structures. In other words, are the well-known protein structure classes indeed recovered from our unsupervised approach (also see, e.g. [16], [64], [65])?

To address this question, we described the structure of each of the 112 proteins by a set of 24 different structure observables (Table 3) such as residual contact matrix, secondary structure content, moments of inertia, and solvent accessible surface (see methods for full details). These 24 structure observables span a structure space with each protein being characterized by one vector, similar to dynamics space. These vectors were then subjected to PCA.

**Table 3:** These 24 structure observables
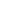
to 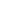 have been used to characterize the protein structure space.

Index	Symbol	Description
1–3	*I_x,y,z_*	Principal moments of inertia
4–6	*φ_x,y,z_*	Dipole moments
7–10	*n^α^* ^,*β*,coil,turn^	Secondary structure content
11,12	*n* ^all,hydrophobic^	Number of intramolecular contacts
13	*n* ^HB^	Number of hydrogen bonds
14,15	sasa,sasa*^hp^*	Solvent accessible surface area
16	*apl*	Average path length
17	*cc*	Cluster coefficient
18	*r*	Cluster radius
19	*n* ^phob^	Number of hydrophobic residues
20	*n* ^phil^	Number of hydrophilic residues
21	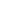	Number of acidic residues
22	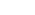	Number of basic residues
23	*n* ^Pro^	Number of proline residues
24	*n* ^Cys^	Number of cysteine residues

**Figure 7:**
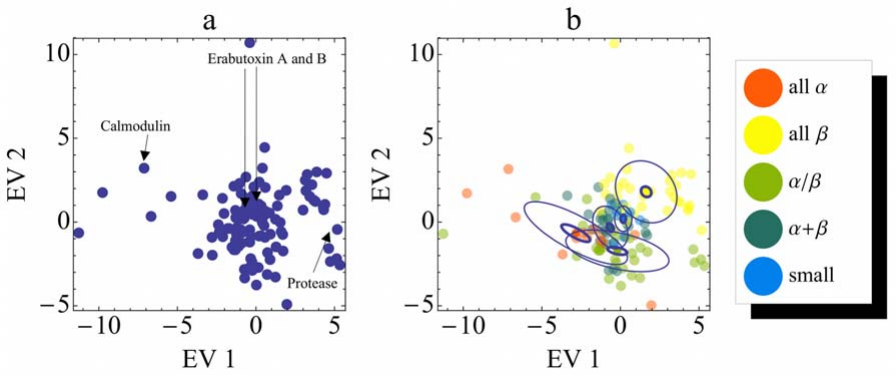
Distribution of proteins in *structure* space. Each point represents one protein. **a)** Protein structures as described by eigenvectors 1 and 2. In plot **a)** the same proteins as in Fig. 3 are labelled. **b)** same projection as in **a)**, but colored according to SCOP structure classes (see legend). Distributions of SCOP classes are described by their standard deviations (thin large ellipses) as well as the standard deviation of their respective means (thick small ellipses).

Figure 7 shows the distribution of protein structures (points) in the plane spanned by the first two eigenvectors obtained from this PCA. As can be seen from Fig. 7a, no clusters are evident in the space of protein structures, quite similar to our observation in the space of protein dynamics. This result supports our above conjecture that SCOP and CATH suggest a much clearer partitioning of protein structure space than is evident from our unsupervised classification from a set of 24 structural observables, and in fact also from other unsupervised approaches [63], [66]. From this point of view, our finding of a rather unstructured dynasome is less surprising.

This result also raises the question if (and how) the positions of proteins in this structure space relate to their respective SCOP classes (Fig. 7b). As can be seen, despite marked overlap of the individual classes (large ellipses) the class centroids are statistically significantly separated (small ellipses). This is a remarkable result *per se*, as it shows that our approach of fully unsupervised structure characterization, which does neither involve sequence information, nor any heuristics, hierarchy, or evolutionary criteria, still recovers the top tier of the hierarchical, manually curated, and evolution-based SCOP classification system.

### Similar Structures May Show Different Dynamics – and Vice Versa

One of the goals of this work is to see if protein dynamics information can be used to improved protein function prediction beyond sequence and structure based schemes [67]. This requires that the dynamics fingerprint considered here holds information which – due to the possibly rather indirect relationship between structure and dynamics – can not be extracted from structures alone. This additional information would show up as incomplete correlation between structure and dynamics. We have therefore quantified this correlation using Euclidean distances in structure and dynamics space, respectively, as an appropriate metric.

In particular, and relating the second of the three scenarios discussed above, this metric will allow to address the question: Given two structurally similar proteins, how similar are their dynamics? Further, does similar structure necessarily imply similar dynamics or, conversely, can similar motion patterns be generated from quite different structures (adjoint dynamics)? Vice versa, can small structure differences imply large differences of protein dynamics (disjoint dynamics)? As above, structural similarity of each protein pair was measured by its Euclidean distance 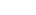 in structure space, and these distances were correlated to their respective counterpart 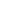 in dynamics space.

**Figure 8:**
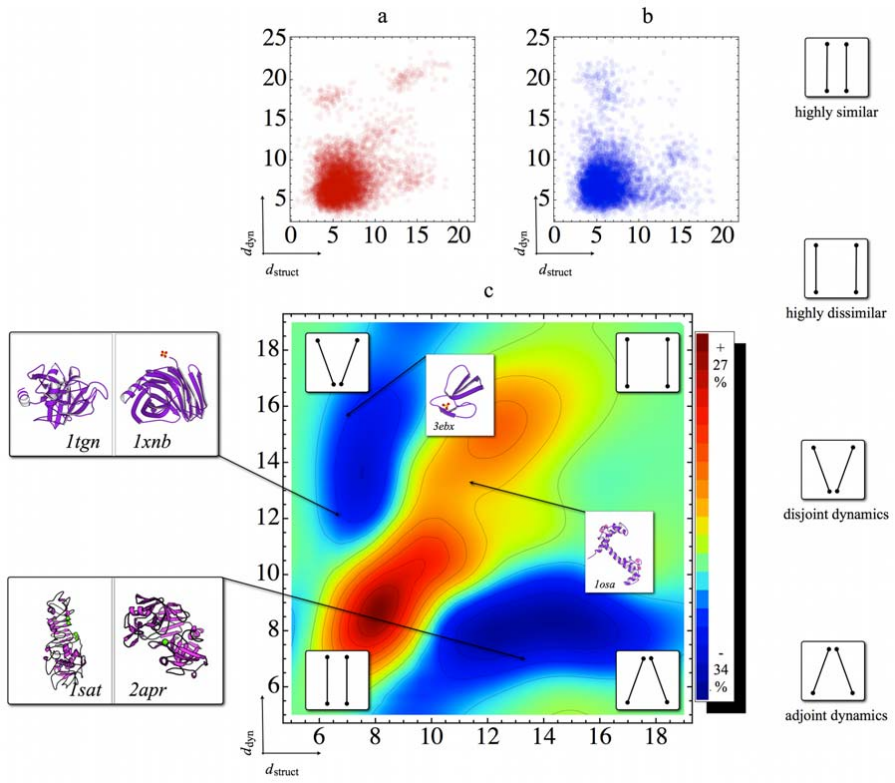
Structure dissimilarity vs dynamics dissimilarity. **a)** Each point displays the structure dissimilarity (*x* axis) vs. the dynamics dissimilarity (*y* axis) for one protein pair. Structure and dynamics dissimilarities for each of the 6216 protein pairs were computed as squared Euclidean distance between any two points in structure and dynamics space, respectively, as described in the text. Distances are unit-less. Regions of larger opacity reflect higher point densities. The overall Pearson correlation coefficient between structure and dynamics is 0.38; **b)** Randomized reference data obtained by removing any correlation between structure and dynamics dissimilarities, as described in methods (cf. Fig. S2); **c)** difference between point densities a) and b), after smoothing with a Gaussian kernel of width 1. Regions of larger than random density are colored red, those of lower density are colored blue, and regions with equal density or no data are shown in green, as quantified by the color bar. Below the diagonal: adjoint dynamics. Above the diagonal: disjoint dynamics. Inset in the red upper right region: Average position of Calmodulin (PDB code 1OSA), which reflects its dissimilarity both in structure and dynamics from most other proteins. Inset in the top of the disjoint dynamics region: Average position of Erabutoxin B (PDB code 3EBX), reflecting its common structure paired with unusual dynamics. Disjoint dynamics region: Position of the pair of trypsin 1TGN and xylanase 1XNB. These two proteins are structurally quite similar but markedly different in dynamics. Adjoint dynamics region: Position of the pair of the hydrolases 1SAT and 2APR, which have dissimilar structure, but display very similar dynamics.

Fig. 7a shows for each of the 6216 protein pairs *i* distances as points in the *x–y* plane. As can be seen, the overall shape does not indicate a particularly strong correlation between structural and dynamics similarities, with a Pearson correlation coefficient of 0.38. This number is difficult to interpret, however, as *a priori* it is unclear how correlations between the *positions* of proteins in high-dimensional dynamics and structure spaces, respectively, relates to the observed correlation between *distances of pairs* of proteins in dynamics space and structure space. In particular, it is unclear whether the observed low correlation coefficient actually implies that our dynamics observables are nearly unrelated to the protein structures. To assess the significance of this correlation, we randomized the coordinates of the dynamics vectors to obtain a reference point cloud (Fig. 8b), which, by construction, lacks any correlation between structure and dynamics (see methods). Figure 8c shows the difference of point densities (color code) between the data in Fig. 8a and the randomized reference data in Fig. 8b. Red regions indicate significantly higher densities than expected for uncorrelated data, blue lower densities, and green indicates regions where dynasome and randomized densities are similar or where no data is available.

The pronounced structure seen in Fig. 8c reveals and quantifies systematic relationships between structure and dynamics, and suggests its classification into four regions, as denoted by four symbols (white insets in the corners). It is, for instance, mainly along the diagonal where significantly more pairs are found than would be expected by chance.

The lower left region contains protein pairs that are similar both in terms of structure *and* dynamics. There is a significant correlation between structural similarity and dynamics similarity beyond what would be expected by chance, as indicated by the coloring. For a small sample of five proteins with similar fold, such correlation has been suggested previously from a coarse grained elastic network analysis [68].

Further along the diagonal, the upper right region contains pairs of protein pairs which are very different in both structure and dynamics. This quadrant is also significantly more populated than expected by chance showing a systematic trend, that structurally different proteins tend to exhibit different dynamics. Calmodulin, whose dynamics and high flexibility are remarkable in many ways (as also reflected by its position in Fig. 4b), also has an unusual structure (Fig. 5a and Fig. 7) different from most other proteins. Accordingly, many pairs involving Calmodulin are located in the indicated region in the top-right corner of the red region of Fig. 8c (inset with PDB code 1OSA).

The two off-diagonal regions (blue), in contrast, indicate structure-dynamics relationships which are underrepresented. The region below the diagonal contains pairs involving proteins with similar dynamics despite dissimilar structure, which we termed “adjoint dynamics”. As an example, consider the two hydrolases with PDB codes 1SAT and 2APR (Fig. 8c, lower left inset), which exhibit similar dynamics despite their quite different structures. The relatively high structural dissimilarity is reflected by high distance in structure space (13.28), and also by undetectable similarity using the pairwise-DALI algorithm [69]. Vice versa, ‘disjoint dynamics’ is seen in the region above the diagonal, where proteins with similar structure display quite different dynamics. Here, the trypsin (1TGN) and the xylanase (1XNB) (Fig. 8c, upper left inset) serve as an example. These two proteins are structurally similar (distance in structure space 6.19), but separated in dynamics space by the large distance of 11.6 units. Comparison of Fig. 8a with c shows that a considerable number of proteins show such remarkable behavior.

The latter two regions are particularly interesting, because they reveal relationships between proteins, which purely structure based classification would miss. Although not derived in an evolutionary context, it is tempting to speculate about the implications of these results. For example, the adjoint dynamics of structurally different proteins may in some cases result from convergent evolution, in cases where similar dynamics is required to achieve a particular function. Conversely, disjoint dynamics may have occurred in response to the need to evolve divergent functionality from a common ancestor. In both cases, one would expect that our analysis of the dynasome should improve protein function predictions.

The dynamics of e.g. Erabutoxin B is quite unusual, in contrast to its unsuspicious structure (Fig. 7). Accordingly, most pairs involving Erabutoxin B are located in the “disjoint dynamics” region above the diagonal. On the one hand, they need rigidity to escape the proteases of the infected immune system; on the other hand they need pronounced flexibility to account for the differences of certain receptors in all the different prey animals they are supposed to attack.

Further below we will give a more systematic account of the relationship between dynamics and function, but first we need to analyze the fine structure of the dynasome.

### Fine Structure of the Dynasome

We have shown above that the dynasome lacks well-separated clusters. Nevertheless, Fig. 4a suggests the existence of some internal substructure, which should reflect the expected relationship between dynamics and function. The *k*-means partitioning employed in Figure 6 might not reveal such fine structure, because it rather focuses at spherically shaped, well-separated regions of high point density and is, furthermore, relatively sensitive to outliers. Therefore, as a complementary approach to *k*-means, we calculated graphs of mutual adjacencies, which are length-scale invariant and do not rely on explicit assumptions about the shape of putative sub-structures. In this approach, two proteins are considered adjacent if they belong to each other’s *k* nearest neighbors in a properly chosen *d*-dimensional subspace of the 34-dimensional dynamics space. The resulting adjacency matrix is represented as a graph, in which each protein is a vertex and two adjacent proteins are connected by an edge. The parameters *d* and *k* were always chosen such that this graph was compact and optimally partitioned, as quantified by the community modularity [70] (see methods).

**Figure 9:**
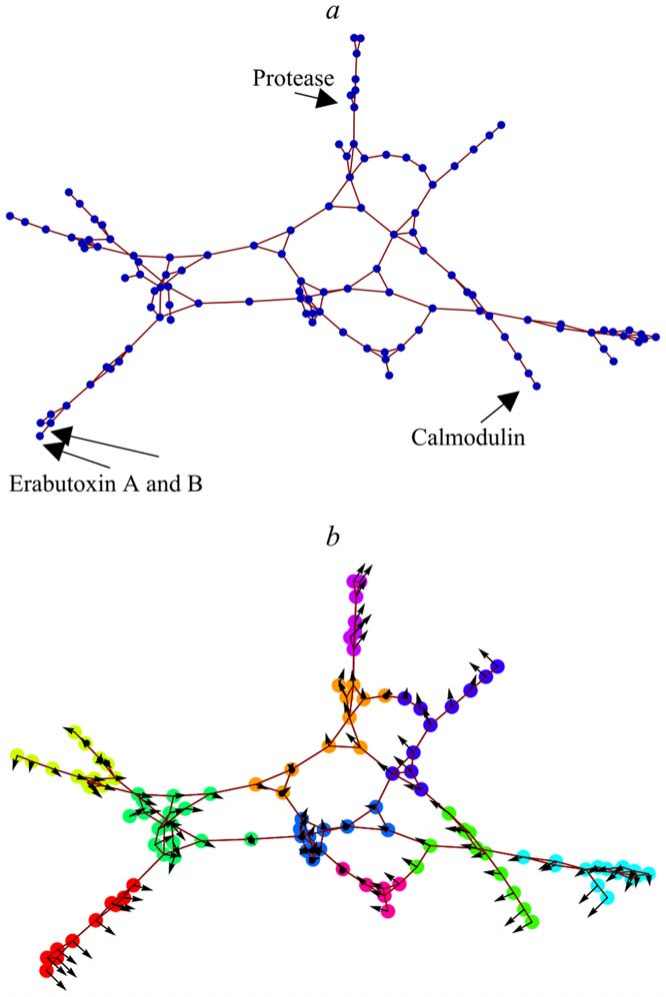
Graph representations of the fine structure of the dynasome. **a)** Graph of the adjacency matrix of dynasome proteins in the space spanned by the first four dynasome descriptors. Vertices represent proteins, edges represent neighborship. Highlighted are proteins discussed in Fig. 4. **b)** same graph as **a)**, but colored according to the clustering obtained by maximizing the community modularity. Arrows indicate the position of each protein on the subspace spanned by the dynasome descriptors 1 and 2 (cf. Fig. 4 and Table 4).

Figure 9a shows the mutual adjacency graph of the dynasome, with 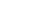 and 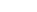 In this representation, proteins with similar dynamics appear as close-by vertices with high connectivity, and clearly separated from other clusters by vertices with relatively low connectivity. Ten clusters (colors) were identified by maximizing the community modularity (Fig. 9b). Comparing Fig. 9b with our previous analyses of the structure of the dynasome shows that this graph is a faithful representation of the dynasome (Text S1 and Fig. S5). In particular, our graph based clusters group proteins with similar positions in the PCA projection (Fig. 4), as indicated by the arrows representing the position of each protein in the plane spanned by dynasome descriptors 1 and 2. Vice versa, additional structure is revealed, as can be seen from the fact that some groups of proteins have almost identical positions in Fig. 4, but are clearly separated in Fig. 9b.

**Table 4:** Composition of the first four dynasome descriptors: Shown are the five observables that contribute most to the first four descriptors; relative contributions to the descriptor are given in percent, (–) indicates that the respective observable appears in the linear combination defining the descriptor with a negative coefficient.

	Descriptor 1	Descriptor 2	Descriptor 3	Descriptor 4
1	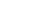 10%	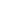 12%	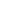 19%	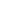 13%
2	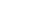 9%	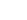 8%	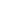 15%	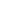 12%
3	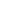 9%	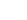 7%	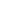 11%	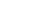 11% (–)
4	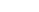 7% (–)	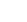 7%	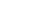 9% (–)	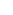 9% (–)
5	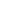 7% (–)	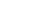 7% (–)	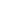 6% (–)	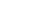 7% (–)

For a list of the symbols, see Table 2. Descriptor 1: Average root mean square fluctuations from the ensemble average 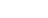 average root mean square deviation from the X-ray structure 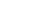 standard deviation (% of mean) of the radius of gyration 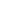 skewness of the ruggedness distribution of the proteins’ degrees of freedom 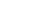 and average ruggedness (averaged over all collective degrees of freedom in the protein) 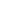 Descriptor 2: friction constants of the diffusion along collective degrees of freedom 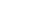 goodness-of-fit of the first principal component to a Gaussian distribution 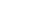 kurtosis of the ruggedness distribution of the proteins’ degrees of freedom 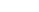 Descriptor 3: Eigenvalues of the protein ensembles’ eigenvectors 5, 1 and 4 (2 and 3 appearing further below and are not explicitly shown here); fluctuation of the RMSD from the X-ray structure 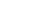 and of the radius of gyration 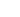 Descriptor 4: Fluctuations of solvent accessible surface, turn content and secondary structure content 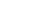 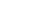 and 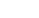

### Function Coins Dynamics

**Figure 10:**
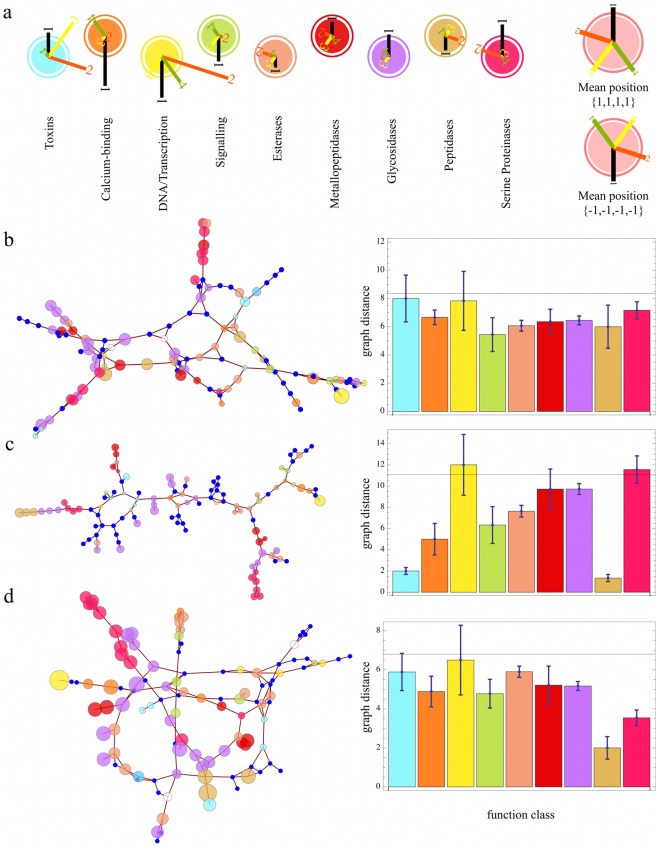
Dynamics fingerprints and relation to function. ** a)** Compass diagrams indicate the average position of each function class in the dynamics space spanned by dynasome descriptors 1–4 (see Table 3 for composition of descriptors). **b–d)** Graphs of adjacency matrices (left) in the dynamics space (**b**), structure space (**c**) and combined space (**d**) and corresponding average distances (right) between proteins of the same function classes (bar heights) vs average distance (solid horizontal lines) between all proteins. The colors denote the protein function classes defined in panel **a**.

In the following we will analyze the correlation between dynamics and function. To this aim we classified the 112 proteins according to literature annotations into 9 relatively broad function classes (see methods and Table S1 for details). We first determined the mean position, or centroid, of each of the 9 function classes in the dynasome space. In Fig. 10a, this average position of each function class is represented by a compass diagram. The lengths (and direction) of the four bars labeled 1,…, 4 correspond to the average position on the first four dynasome descriptors. If function classes were randomly distributed, the mean position of each class would fall onto the origin. Instead, we find that each function class has its unique dynamics fingerprint. For example, as indicated by descriptor 1 (black) in the compass plots, esterases (centre, light orange) appear to sample a smoother free energy landscape, according to Table 4, than that of glycosidases (purple), where the respective projection has an opposite sign. Also, esterases tend to fluctuate at slower time scales (descriptor 2 compass plots, cf. Table 4). In contrast, these two functional groups show similar collectivity of the functional modes (descriptor 3, cf. Table 4) and fluctuations of secondary structure elements (descriptor 4). The most flexible proteins in our set are calcium-binding proteins (deep orange). These exhibit the most pronounced secondary structure fluctuations (descriptor 4) and in that respect differ strongly from typical DNA/transcription related proteins (yellow), which in turn fluctuate on the fastest time scales of all examined proteins.

These pronounced differences in the average positions, to which we will refer to as ‘dynamics fingerprint’ of the proteins, should also show up in the graph representations of the dynamics space. Fig. 10b (left; see also Fig. S6 left column), reproduces the dynamics graph introduced in Fig. 9 here with the nodes colored according to their function classification. As can be seen, the clustering identified in Fig. 9, although purely based on dynamics descriptors, reflects the functional classification shown in Fig. 10b (left) to a remarkable extent. For instance, three out of four DNA/transcription related proteins (yellow) are on the rightmost branch, almost all proteins in the top-left branch are glycosidases (purple), and serine proteinases (magenta) dominate the top branch and the lower left branch.

This first visual impression was quantified by comparing the average distance of any two proteins of the same function to the average distance of all protein pairs. Fig. 10b (right) shows the mutual average distance in the graph for each function class (bar heights), i.e. the number of edges connecting two proteins of the same function, as well as the standard deviation of that average. As can be seen, proteins of the same function class are, overall, significantly closer to one another than the average distance of all vertices in the graph (black horizontal line), which one would expect in the absence of any correlation between dynamics and function. Exceptions are the classes DNA/Transcription and Toxins.

The fact that functionally similar proteins occupy similar regions of the dynamics space suggests a straightforward way to infer function purely from dynamics similarity by partitioning the dynamics space into ‘function neighborhoods’, i.e. areas of the dynamics space which are occupied preferentially by a given function class. Accordingly, the centroids of these function neighborhoods serve as the reference points, and proteins of unknown function are then predicted to share the function of their closest neighborhood (the function class with the lowest average graph distance). To quantify the predictive power of this approach, we determined if this procedure would have predicted the correct function class. To that aim, the shortest path from each protein to all other proteins of known function was calculated. We then checked whether its known function corresponds to the function of the closest function class, in which case we would have obtained a correct assignment. Remarkably, correct assignments were found in 57% of all cases, as compared to 11% expected in the absence of any correlation between dynamics and function. A detailed comparison by function class (Table S3) suggests different success rates for the different function classes. The dynamics fingerprint of glycosidases (12 correct assignments out of 17) seems to be quite characteristic for this function class, whereas esterases dynamics turned out to be more diffuse, allowing only 4 out of 13 proteins to be assigned correctly.

For rigorous cross-validation, the above algorithm was modified such that for each protein assumed unknown, the function centroid was re-calculated from the remaining proteins only. As above, the predictive power was then assessed via similarity of the functional class of each protein with that of the closest centroid. Focussing at the three largest function groups for which sufficient proteins (>10) are available to obtain reasonable statistical accuracy, correct assignments were obt’ained in 46% of all cases (Table S3), which is only slightly below the above correlation, thus establishing remarkable predictive power of this simple approach. For the remaining and quite small function classes, a value of only 7% is obtained due to poor statistics, such that predictive power is not established for these classes.

Having established a clear dynamics function correlation we next examined whether, with a similar approach, similar correlations are seen between structure and function. To this end, we computed the adjacency matrix of proteins in structure space. The resulting graph is shown in Fig. 10c (left; and Fig. S6, centre column). Similar to dynamics space, local accumulations of function classes can be seen, also yielding significantly lower than average intra-class distances for most function classes (bar plot Fig. 10c right). This observation corroborates the well-known fact that structural alignments can in many cases improve protein function predictions [7].

With an approach similar to that applied to the dynamics fingerprints above, we found a structure/function correlation of 36%, smaller than the dynamics/function correlation above (Table S3). The cross-correlation test yields 27% correct annotations for the three larger groups, and 39% for the remainder. The overall prediction rate of 32% agrees well with published structure based prediction rates [7]. Similar to the prediction based on dynamics, cross-validated prediction rates are only 2% smaller than the observed correlations for the three largest function classes, but drop by 8% for the small groups for which the statistics is poor.

Would one expect to improve the prediction rate even further by using both, structure and dynamics information? At first sight, this should be the case, particularly for those function classes, which form relatively compact clusters in dynamics space, but are structurally quite unrelated (e.g., calcium binding proteins). In these cases, a better prediction rate is expected for a purely dynamics based function prediction than for a purely structure based one. However, other function classes (e.g., peptidases) appear to form compact clusters in structure but not in dynamics space. For these classes, structure based predictions should be superior. Due to this complementarity, it is not clear *a priori* whether or not the combined use of structure and dynamics actually will improve function prediction rates.

To resolve this issue, we combined our structure and dynamics space into a 34+24 = 58 dimensional space. Similarly to the above procedure, a PCA on the respective combined descriptors was carried out, and adjacency relations in the space spanned by the first five 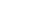 collective descriptors were obtained from the PCA. Note that this 5-dimensional subspace does not necessarily weight dynamics and structural features equally.

Figure 10d (left) shows the resulting graph of the calculated adjacency matrix. Analysis of the underlying adjacency matrix shows that proteins of common function class are, on average, closer to each other in this combined space than they are in dynamics and structure space alone (Fig. 10d right; and Fig. S6, right column), whereas the average distance in this graph is also markedly smaller. Using both structure and dynamics information, 35% correct annotations are achieved, slightly more than structure and dynamics based predictions alone. We note that more elaborate combinations of structure and dynamics space, and optimized choices of *k* and *d* are likely to further improve the predictive value of our approach.

### Conclusions

Inspired by the successful classifications of the sequence and structure space covered by proteins, we investigated the *dynamics space* covered by small, soluble proteins chosen from many structural families and folds. The dynamics of each protein was characterized by a set of 34 dynamics descriptors spanning the dynamics space. We referred to the whole set of protein dynamics patterns as the dynasome. Remarkably, the grand distinction between thermodynamics and kinetics properties of a many-particle system was found to be already encoded, in terms of the directions of its two largest extensions, within the structure of the dynasome.

The first question we addressed was whether or not these proteins naturally fall into dynamics classes, such as seen for the more established sequence and structure classifications. We found that proteins populate the dynamics space continuously, and no canonical partitioning, which would enable an unambiguous classification, was seen. The observation that functionally unusual proteins appear as outliers in this dynamics space provided a first hint towards a close connection between function and dynamics. A systematic analysis of the dynasome indeed revealed remarkably large correlations between dynamics and protein function. Functional classes leave distinct “fingerprints” in the dynasome, which we were able to characterize using only a few collective dynasome descriptors. The finding that proteins of similar function cluster in dynamics space led us to a new and straightforward protein function prediction approach, purely based on protein dynamics similarity. Indeed, already for the relatively small set of proteins considered here, such an approach yielded correct annotations for 46% of the largest functional classes, which is comparable to the performance of the most advanced structure based methods.

A second set of questions addressed was how protein structure relates to protein dynamics and, in particular, to which extent structurally similar proteins exhibit similar dynamics. We characterized protein structure using the same unsupervised approach as for protein dynamics. In this case, protein structure was represented by a 24 dimensional vector of structure descriptors. The structural similarity between any two proteins was then quantified by the distance in structure space, different from the usual approach based on RMSDs between subsets of atoms (e.g. C^α^ atoms) according to some domain hierarchy.

As one might expect, many structurally similar proteins were found to exhibit similar dynamics and, vice versa, many structurally different proteins tend to perform different dynamics, thus establishing significant structure-dynamics correlation. In a significant number of cases, however, this straightforward structure-dynamics relation was found to be violated. Quite different structures shared similar motion patterns (‘adjoint’), and other very similar structures exhibited quite different dynamics (‘disjoint’). In these cases, dynamics relations offer a viewpoint that is complementary to that derived from structural characterizations.

To decide which of the two views is more closely linked to protein function, we also investigated how well function can be determined purely from structure within our framework and obtained a success rate of 32%. Combining structure and dynamics information yielded an intermediate rate of 35%, a slightly higher value than either dynamics or structure based predictions alone. It seems likely, that prediction methods can be devised which combine the information from structure and dynamics in a more elaborate manner, and thus enable even more accurate predictions, e.g. optimizing the parameters *d* and *k* for this specific purpose. The findings presented in this study are remarkable in the light of the fact that our dynamics descriptors, being derived from 100 ns simulations, provide a quite limited “window” to the full dynamics. In particular slow dynamics are entirely missed, as are other dynamics features that are not captured by our observables. Despite this fact, however, our limited view seems to suffice, to predict protein function at a remarkable rate, and therefore captures functionally relevant parts of the dynamics. By analogy, to identify a murderer, one does not always require a photograph of his whole body, often a fingerprint suffices. Here, we presented fingerprints of protein dynamics.

## Supporting Information

Figure S1 **Observable correlation.** Pairwise absolute Pearson’s correlation coefficients (color codes see legend) between the dynamics observables used in this study. Observables indices correspond to main Table 2.
(TIFF)

Figure S2 **Principle of decorrelation between two arbitrary variables** ***x*** **and** ***y*** **.** The correlation seen in (a) is removed by applying a random permutation to the *y*-component (b).
(TIFF)

Figure S3 **Parameter optimization for mutual adjacency graphs.** The *k* nearest neighbors, which define the connectivity of each protein in the *d* dimensional subspace of the a) dynamics, b) structure, c) combined dynamics and structure space. The partitioning of each resulting graph for each pair {*k,d*} is quantified by the community modularity *C* (z-axis). For the subsequent analyses, *k* and *d* were chosen such that *C* was maximized (black points).
(TIFF)

Figure S4
**Determination of natural partitioning of the dynasome.** Average Connectivity (x-axis) vs Average Variance (y-axis) for *k*-means partitioning into 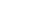 clusters (numbers). For the optimal number of clusters, both measures are minimal. For the dynasome, no such optimal number could be identified.(TIFF)

Figure S5 **Graph of a adjacency matrix of dynasome proteins in the dynamics space.** Vertex colours indicate (a) k-means clusters in the whole 34-dimensional dynamics space (same clusters like main text Fig. 6b), (b) SCOP classes of proteins.
(TIFF)

Figure S6 **Co-location of proteins of the same functional class distinct functional classes in the neighborhood plot of the dynamics space (left column), structure space (middle column), and combined dynamics and structure space (right column).** Colors indicate function classes according to the colour code in main text Fig. 10.
(TIFF)

Table S1 **Systems (PDB codes) selected for analysis.** Functions were obtained as described in methods. Poorly covered function classes were assigned “Other” and “Other Enzymes” and not included in the graph analyses.
(TEX)

Table S2
**Fit functions for Sequence length decorrelation of dynamic (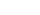) and structure (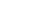) observables.**
(TEX)

Table S3 **Prediction rate by function class.** Numbers in brackets indicate the result of a cross validation test, described in the main text.
(TEX)

TEXT S1 **The graphs are a faithful map of the dynasome.**
(PDF)

## References

[1] Anfinsen C, Haber E (1961). Studies on the reduction and re-formation of protein disulfide bonds.. J Biol Chem.

[2] Erdin S, Lisewski AM, Lichtarge O (2011). Protein function prediction: towards integration of similarity metrics.. Curr Opinion Struct Biol.

[3] Chothia C, Lesk A (1986). The relation between the divergence of sequence and structure in proteins.. EMBO J.

[4] Sander C, Schneider R (1991). Database of homology-derived protein structures and the structural meaning of sequence alignment.. Proteins.

[5] Wilson C, Kreychman J, Gerstein M (2000). Assessing annotation transfer for genomics: Quantifying the relations between protein sequence, structure and function through traditional and probabilistic scores.. J Mol Biol.

[6] Devos D, Valencia A (2000). Practical limits of function prediction.. Proteins.

[7] Whisstock J, Lesk A (2003). Prediction of protein function from protein sequence and structure.. Q Rev Biophys.

[8] Altschul S, Gish W, Miller W, Myers E, Lipman D (1990). Basic Local Alignment Search Tool.. J Mol Biol.

[9] Altschul S, Madden T, Schaffer A, Zhang J, Zhang Z (1997). Gapped BLAST and PSI-BLAST: A new generation of protein database search programs.. Nucleic Acids Res.

[10] Skolnick J, Kolinski A, Kihara D, Betancourt M, Rotkiewicz P (2002). Ab initio protein structure prediction via a combination of threading, lattice folding, clustering, and structure refinement.. Proteins.

[11] Jones D, Tress M, Bryson K, Hadley C (1999). Successful recognition of protein folds using threading methods biased by sequence similarity and predicted secondary structure.. Proteins.

[12] Hildebrand A, Remmert M, Biegert A, Söding J (2009). Fast and accurate automatic structure prediction with HHpred.. Proteins.

[13] von Grotthuss M, Plewczynski D, Vriend G, Rychlewski L (2008). 3D-Fun: predicting enzyme function from structure.. Nucleic Acids Res.

[14] Pearl F, Bennett C, Bray J, Harrison A, Martin N (2003). The CATH database: an extended protein family resource for structural and functional genomics.. Nucleic Acids Res.

[15] Andreeva A, Howorth D, Brenner S, Hubbard T, Chothia C (2004). SCOP database in 2004: refinements integrate structure and sequence family data.. Nucleic Acids Res.

[16] Holm L, Sander C (1996). Mapping the protein universe.. Science.

[17] Pascual-Garcia A, Abia D, Mendez R, Nido GS, Bastolla U (2010). Quantifying the evolutionary divergence of protein structures: The role of function change and function conservation.. Proteins.

[18] Perutz M (1970). Stereochemistry of cooperative effects in haemoglobin.. Nature.

[19] Ansari A, Berendzen J, Bowne S, Frauenfelder H, Iben I (1985). Protein states and proteinquakes.. Proceedings of the National Academy of Sciences.

[20] de Groot B, Grubmuller H (2001). Water permeation across biological membranes: Mechanism and dynamics of aquaporin-1 and glpf.. Science.

[21] Pang A, Arinaminpathy Y, Sansom M, Biggin P (2005). Comparative molecular dynamics - similar folds and similar motions?. Proteins.

[22] Yaneva R, Springer S, Zacharias M (2009). Flexibility of the MHC class II peptide binding cleft in the bound, partially filled, and empty states: A molecular dynamics simulation study.. Biopolymers.

[23] Cox K, Sansom M (2009). One membrane protein, two structures and six environments: a comparative molecular dynamics simulation study of the bacterial outer membrane protein pagp.. Mol Membr Biol.

[24] Meyer T, de la Cruz X, Orozco M (2009). An atomistic view to the gas phase proteome.. Structure.

[25] Jonsson AL, Scott KA, Daggett V (2009). Dynameomics: A consensus view of the protein unfolding/folding transition state ensemble across a diverse set of protein folds.. Biophys J.

[26] Toofanny RD, Jonsson AL, Daggett V (2010). A comprehensive multidimensionalembedded, one-dimensional reaction coordinate for protein unfolding/folding.. Biophys J.

[27] van der Kamp MW, Schaeffer RD, Jonsson AL, Scouras AD, Simms AM (2010). Dynameomics: A comprehensive database of protein dynamics.. Structure.

[28] Meyer T, D’Abramo M, Hospital A, Rueda M, Ferrer-Costa C, et al. (2010). MoDEL (Molecular Dynamics Extended Library): A database of atomistic molecular dynamics trajectories.. Structure.

[29] Shaw DE (2009). Anton: A specialized machine for millisecond-scale molecular dynamics simulations of proteins.. Abstr Pap Am Chem S.

[30] Shaw D, Maragakis P, Lindorff-Larsen K, Piana S, Dror R (2010). Atomic-level characterization of the structural dynamics of proteins.. Science.

[31] Amadei A, Linssen A, Berendsen H (1993). Essential dynamics of proteins.. Proteins.

[32] Zen A, Carnevale V, Lesk AM, Micheletti C (2008). Correspondences between lowenergy modes in enzymes: Dynamics-based alignment of enzymatic functional families.. Protein Sci.

[33] Munz M, Lyngso R, Hein J, Biggin P (2010). Dynamics based alignment of proteins: an alternative approach to quantify dynamic similarity.. BMC Bioinformatics.

[34] Hooft R, Sander C, Scharf M, Vriend G (1996). The PDBFINDER database: a summary of PDB, DSSP and HSSP information with added value.. Bioinformatics.

[35] Henrick K, Thornton J (1998). PQS: a protein quaternary structure file server.. Trends Biochem Sci.

[36] Hooft R, Vriend G, Sander C, Abola E (1996). Errors in protein structures.. Nature.

[37] Vriend G (1990). WHAT IF: a molecular modeling and drug design program.. J Mol Graph.

[38] Haas J, Lange O, Vriend G, de Groot B, Grubmuller H WHAG – GROMACS interface to WHATIF..

[39] Hooft R, Sander C, Vriend G (1996). Positioning hydrogen atoms by optimizing hydrogen-bond networks in protein structures.. Proteins.

[40] Spoel DVD, Lindahl E, Hess B, Groenhof G, Mark A (2005). GROMACS: Fast, flexible, and free.. J Comp Chem.

[41] Jorgensen W, Tiradorives J (1988). The OPLS potential functions for proteins – Energy minimizations for crystals of cyclic peptides and crambin.. J Am Chem Soc.

[42] Berendsen H, Postma J, van Gunsteren W, DiNola A, Haak J (1984). Molecular dynamics with coupling to an external bath.. J Chem Phys.

[43] Hess B, Bekker H, Berendsen H, Fraaije J (1997). LINCS: A linear constraint solver for molecular simulations.. J Comp Chem.

[44] Miyamoto S, Kollman P (1992). SETTLE - An analytical version of the SHAKE and RATTLE algorithm for rigid water models.. J Comp Chem.

[45] Darden T, York D, Pedersen L (1993). Particle Mesh Ewald - an N.Log(N) method for Ewald sums in large systems.. J Chem Phys.

[46] Hess B (2000). Similarities between principal components of protein dynamics and random diffusion.. Phys Rev E.

[47] Uhlenbeck GE, Ornstein LS (1930). On the theory of the Brownian motion.. Phys Rev.

[48] Zwanzig R (1988). Diffusion in a rough potential.. P Natl Acad Sci Usa.

[49] Kabsch W, Sander C (1983). Dictionary of protein secondary structure – patternrecognition of hydrogen-bonded and geometrical features.. Biopolymers.

[50] Case DA, Darden TA, Cheatham TE, Simmerling CL, Wang J (2008). AMBER 10, University of California, San Francisco.

[51] Hubbard, Thornton J (1993). NACCESS - atomic solvent accessible area calculations – computer program.. http://www.bioinf.manchester.ac.uk/naccess/.

[52] Hess B, Kutzner C, van der Spoel D, Lindahl E (2008). GROMACS 4: Algorithms for highly efficient, load-balanced, and scalable molecular simulation.. J Chem Theory Comput.

[53] Ahmad S, Sarai A (2011). Analysis of electric moments of RNA-binding proteins: implications for mechanism and prediction.. BMC Structural Biology.

[54] Vendruscolo M, Dokholyan N, Paci E, Karplus M (2002). Small-world view of the amino acids that play a key role in protein folding.. Phys Rev E.

[55] Seber GAF (1984). Cluster Analysis, in Multivariate Observations, John Wiley & Sons, Inc., Hoboken, NJ, USA.

[56] Bairoch A, Bougueleret L, Altairac S, Amendolia V, Auchincloss A (2008). The universal protein resource (uniprot).. Nucleic Acids Research.

[57] Berman H, Westbrook J, Feng Z, Gilliland G, Bhat T (2000). The protein data bank.. Nucleic Acids Res.

[58] Kitao A, Gō N (1991). Conformational dynamics of polypeptides and proteins in the dihedral angle space and in the cartesian coordinate space: Normal mode analysis of deca-alanine.. J Comput Chem.

[59] Kitao A, Go N (1999). Investigating protein dynamics in collective coordinate space.. Curr Opinion Struct Biol.

[60] Handl J, Knowles J, Kell D (2005). Computational cluster validation in post-genomic data analysis.. Bioinformatics.

[61] Sadreyev R, Kim BH, Grishin N (2009). Discrete-continuous duality of protein structure space.. Current opinion in structural biology.

[62] Skolnick J, Arakaki A, Lee S, Brylinski M (2009). The continuity of protein structure space is an intrinsic property of proteins.. Proceedings of the National Academy of Sciences.

[63] Pascual-Garcia A, Abia D, Ortiz AR, Bastolla U (2009). Cross-over between discrete and continuous protein structure space: Insights into automatic classification and networks of protein structures.. PLoS Comput Biol.

[64] Holm L, Sander C (1997). New structure – novel fold?. Structure.

[65] Hou J, Jun SR, Zhang C, Kim SH (2005). Global mapping of the protein structure space and application in structure-based inference of protein function.. Proc Natl Acad Sci USA.

[66] Sadowski M, Taylor W (2010). On the evolutionary origins of "fold space continuity": A study of topological convergence and divergence in mixed alpha-beta domains.. J Struct Biol.

[67] Lisewski A, Lichtarge O (2006). Rapid detection of similarity in protein structure and function through contact metric distances.. Nucleic Acids Res.

[68] Keskin O, Jernigan R, Bahar I (2000). Proteins with similar architecture exhibit similar large-scale dynamic behavior.. Biophys J.

[69] Hasegawa H, Holm L (2009). Advances and pitfalls of protein structural alignment.. Curr Opinion Struct Biol.

[70] Clauset A (2005). Finding local community structure in networks.. Phys Rev E.

[71] Ban C, Ramakrishnan B, Ling K, Kung C, Sundaralingam M (1994). Structure of the recombinant paramecium-tetraurelia calmodulin at 1.68 angstrom resolution.. Acta Crystallogr D.

[72] Smith J, Corfield P, Hendrickson W, Low B (1988). Refinement at 1.4 Å resolution of a model of erabutoxin-B - treatment of ordered solvent and discrete disorder.. Acta Crystallogr A.

[73] Tsunawasa S, Masaki T, Hirose M, Soejima M, Skaiyama F (1989). The primary structure and structural characteristics of achromobacter-lyticus protease-I, a lysinespecific serine protease.. J Biol Chem.

[74] Saarinen M, Gleason F, Eklund H (1995). Crystal structure of thioredoxin-2 from Anabaena.. Structure.

[75] Papageorgiou A, Tranter H, Acharya K (1998). Crystal structure of microbial superantigen staphylococcal enterotoxin B at 1.5 Å resolution: implications for superantigen recognition by mhc class ii molecules and t-cell receptors.. J Molecular Biology.

[76] Acharya K, Shapiro R, Riordan J, Vallee B (1995). Crystal-structure of bovine angiogenin at 1.5-angstrom resolution.. P Natl Acad Sci USA.

[77] Kraulis P (1991). MOLSCRIPT: a program to produce both detailed and schematic plots of protein structures.. J App Cryst.

