# Supplementary figures and images for: Exploring Protein Dynamics Space: The Dynasome as the Missing Link between Protein Structure and Function
Source: PLoS One. 2012 May 11;7(5):e33931. doi: 10.1371/journal.pone.0033931 (PMC3350514; doi:10.1371/journal.pone.0033931)

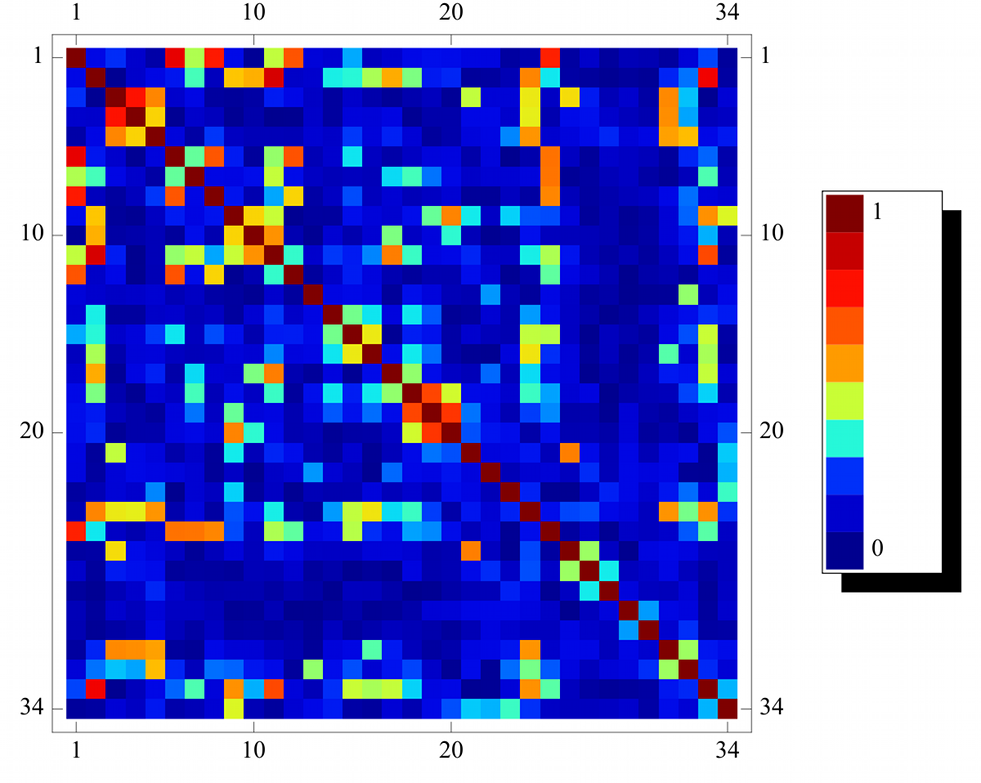

Supplement: Figure S1 — Observable correlation. Pairwise absolute Pearson’s correlation coefficients (color codes see legend) between the dynamics observables used in this study. Observables indices correspond to main Table 2. (TIFF) [file pone.0033931.s001.tif]

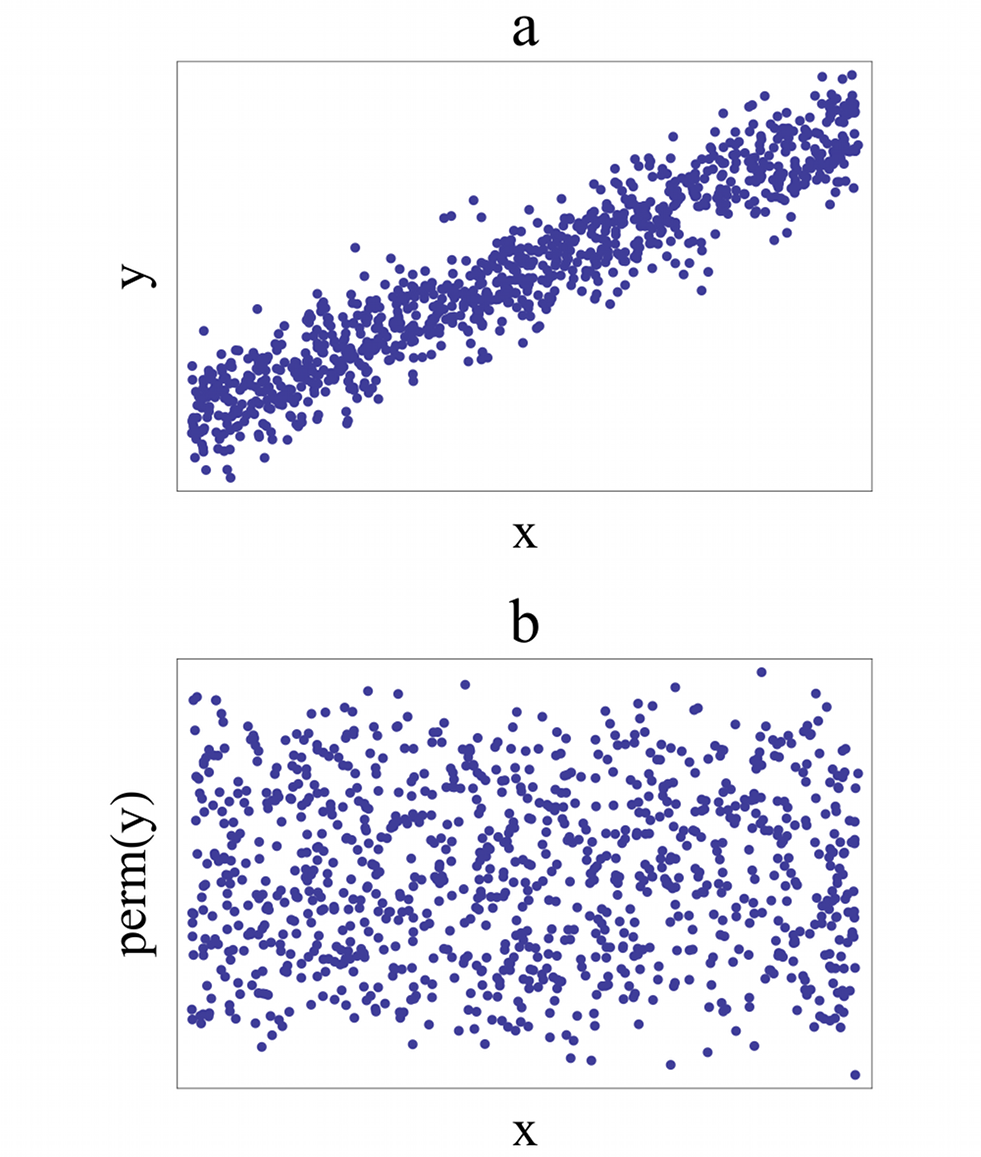

Supplement: Figure S2 — Principle of decorrelation between two arbitrary variables x and y . The correlation seen in (a) is removed by applying a random permutation to the y-component (b). (TIFF) [file pone.0033931.s002.tif]

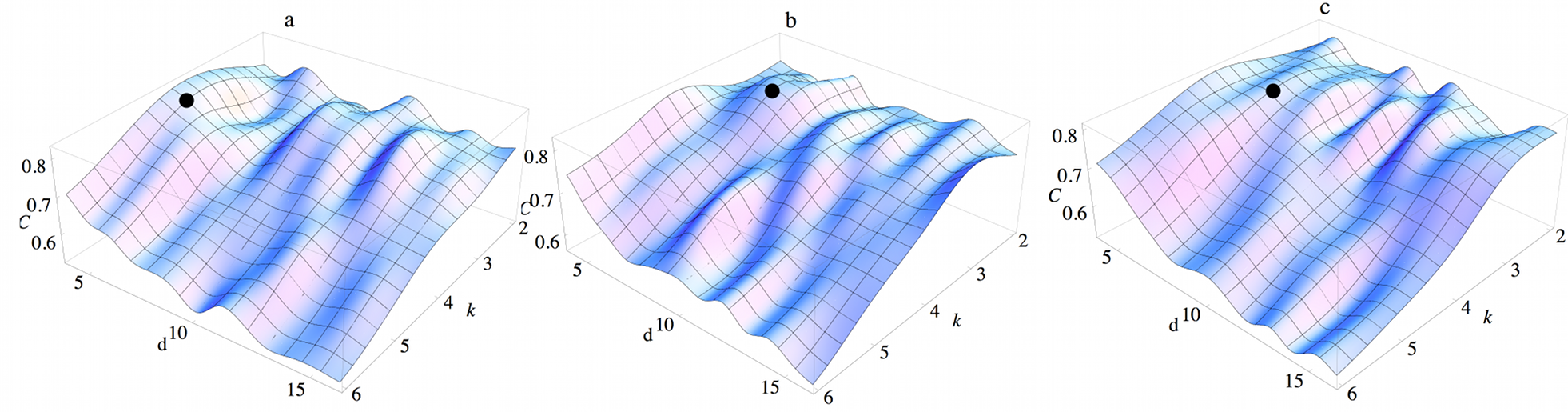

Supplement: Figure S3 — Parameter optimization for mutual adjacency graphs. The k nearest neighbors, which define the connectivity of each protein in the d dimensional subspace of the a) dynamics, b) structure, c) combined dynamics and structure space. The partitioning of each resulting graph for each pair {k,d} is quantified by the community modularity C (z-axis). For the subsequent analyses, k and d were chosen such that C was maximized (black points). (TIFF) [file pone.0033931.s003.tif]

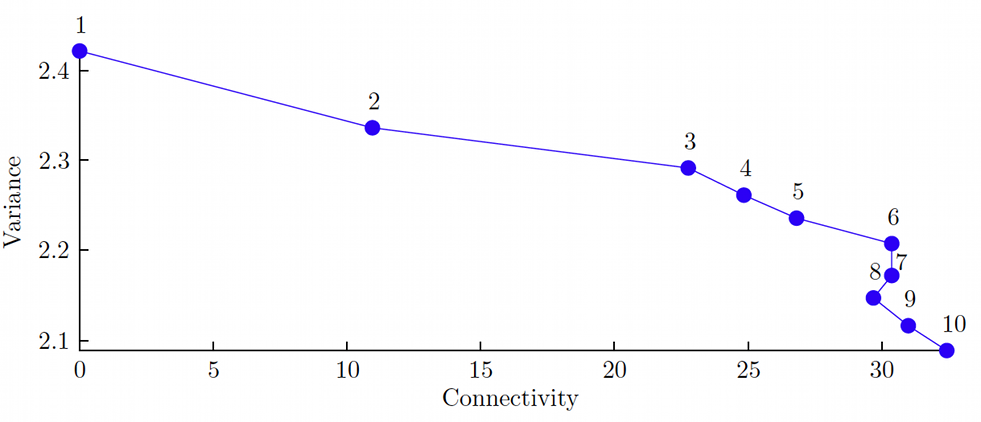

Supplement: Figure S4 — Determination of natural partitioning of the dynasome. Average Connectivity (x-axis) vs Average Variance (y-axis) for k-means partitioning into clusters (numbers). For the optimal number of clusters, both measures are minimal. For the dynasome, no such optimal number could be identified. (TIFF) [file pone.0033931.s004.tif]

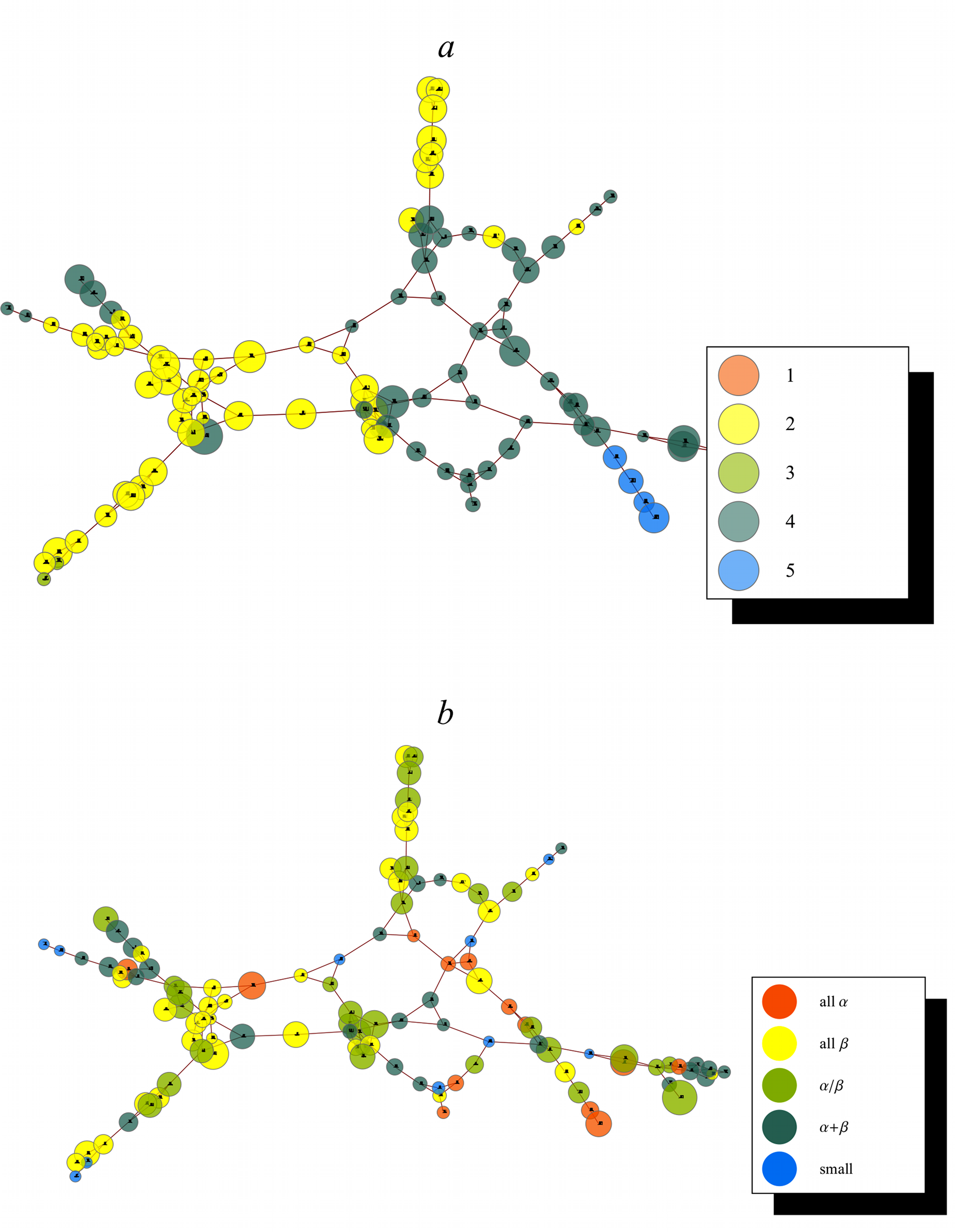

Supplement: Figure S5 — Graph of a adjacency matrix of dynasome proteins in the dynamics space. Vertex colours indicate (a) k-means clusters in the whole 34-dimensional dynamics space (same clusters like main text Fig. 6b), (b) SCOP classes of proteins. (TIFF) [file pone.0033931.s005.tif]

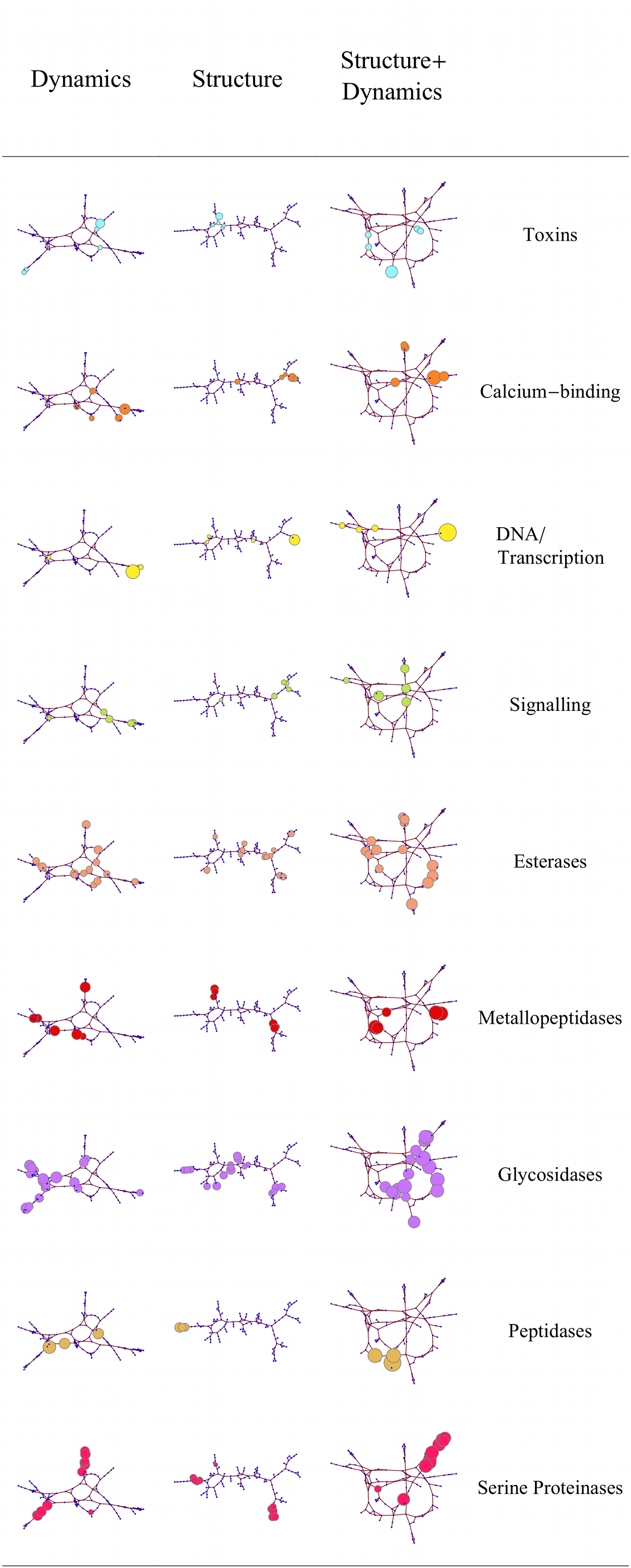

Supplement: Figure S6 — Co-location of proteins of the same functional class distinct functional classes in the neighborhood plot of the dynamics space (left column), structure space (middle column), and combined dynamics and structure space (right column). Colors indicate function classes according to the colour code in main text Fig. 10. (TIFF) [file pone.0033931.s006.tif]
