# Supplementary material for: Exploring Protein Dynamics Space: The Dynasome as the Missing Link between Protein Structure and Function
Source: PLoS One. 2012 May 11;7(5):e33931. doi: 10.1371/journal.pone.0033931 (PMC3350514; doi:10.1371/journal.pone.0033931)
Supplement: TEXT S1 — The graphs are a faithful map of the dynasome. (PDF) [file pone.0033931.s010.pdf]

The graphs are a faithful map of the dynasome

First, to make contact with the entirely PCA-based visualisations, the position of each protein in the plane spanned by dynasome descriptors 1 and 2 (cf. main text Fig. 4) is indicated as an arrow overlying the protein's vertex in Fig. 9b. As can be seen, most proteins of the same cluster have similar positions in the plane of dynasome descriptor 1 and 2. Exceptions to this general observation are, to a different degree in different clusters, rather found close to the cluster borders, where proteins already tend to assume intermediate positions between the typical positions of the two neighbouring clusters. A second observation from Fig. 9b, which can also be derived from the arrows, is that different clusters may have almost identical positions in Fig. 4.

Thus, the information conveyed by dynasome descriptors 1 and 2 are not masked by the higher dimensions, because clusters of proteins identified in Fig. 9b are fairly consistent in terms of positions on the plane of these descriptors 1 and 2. On the contrary, the properties described by higher descriptors allow for a clear discrimination between proteins which almost overlie each other on the plane of descriptors 1 and 2, i.e. which are almost identical in terms of ruggedness, overall flexibility and typical timescales. This is visible by the fact that even distant clusters in Fig. 9b are almost identical in terms of descriptors 1 and 2 (arrows). From Fig. 9 it is also evident that, as speculated before, the reasons for the significant point overlap in Fig. 4 is indeed the fact that descriptors 1 and 2 do not characterize all the different aspects of protein dynamics in sufficient detail to descriminate clearly between classes of protein dynamics. More dynasome descriptors are needed to this end, and further analyses showed that the first four dynasome descriptors are sufficient to uniquely describe all clusters.

As a second test, to compare this result with our previous k-means partitioning of the dynamics space, we mapped the k-means clusters into the graphs (Fig. S5a). As can be seen, the k-means partitioning does not contradict our graph partitions, but is much more coarse-grained and not suitable to resolve the fine structure of the dynamics space. Thus, it is likely that the k-means structure/dynamics correlation analysis (Fig. 6) can be improved by comparing the positions of the proteins in the graph (Fig. 9) with their structure class.

Fig. S5b has the vertices coloured according to the protein's SCOP class. The comparison of Fig. S5a with Fig. S5b shows that indeed the k-means clusters do not find clear-cut separations between structure classes, because there is no such obvious separation but at most an accumulation of all-β and α/β proteins on the l.h.s. of the graph, a fact which is described by the k-means partitioning.

Interestingly however, adjoint/disjoint dynamics are also visible in Fig. S5b from close-by/distant vertices of different/equal colour.

Taken together, these checks suggest that the graphs of the dynasome are indeed a faithful representation of the structure of our dynamics space.
